# Polysaccharide Reengineering: Strategic Modifications and Biofunctional Outcomes of Natural Polysaccharides

**DOI:** 10.3390/ph19101596

**Published:** 2026-10-09

**Authors:** Alka Lohani, Ajita Paliwal, Arti Gautam, Ana Figueiras, Filipa Mascarenhas-Melo

**Affiliations:** 1 Amity Institute of Pharmacy, Amity University, Noida 201313, Uttar Pradesh, India; 2 SBS Group of Educational and Training Institutes, College of Pharmacy, Fatehpur 212664, Uttar Pradesh, India; 3 Laboratory of Drug Development and Technologies, Faculty of Pharmacy, University of Coimbra, 3000-548 Coimbra, Portugal; rfigueiras@ff.uc.pt; 4 REQUIMTE/LAQV, Group of Pharmaceutical Technology, Faculty of Pharmacy, University of Coimbra, 3000-548 Coimbra, Portugal; 5 Higher School of Health, University of Algarve, Campus de Gambelas, 8005-139 Faro, Portugal; 6 BRIDGES—Biotechnology Research, Innovation and Design for Health Products, Polytechnic University of Guarda, 6300-559 Guarda, Portugal

**Keywords:** polymer, polysaccharide modification, chemical modification, grafting, biological activity, pharmaceutical applications

## Abstract

Natural polysaccharides have attracted considerable interest in pharmaceutical and biomedical applications. However, native polysaccharides often possess limitations, like poor solubility, inadequate mechanical strength, limited stability, and insufficient control over their functional and biological properties. Consequently, structural modification and function-driven reengineering of natural polysaccharides have emerged as important strategies for tailoring the physicochemical and biological characteristics to meet the requirements of advanced pharmaceutical and biomedical systems. Beyond individual modification strategies, polysaccharide reengineering provides a design-oriented framework in which structural features are deliberately tuned to establish relationships between molecular or network architecture, physicochemical properties, biointerfacial interactions, and biological function. This review examines polysaccharide reengineering as a function-driven approach to tailoring the structure and performance of natural polysaccharides. Rather than simply cataloguing modification methods, the review critically connects principal reengineering strategies, including carboxymethylation, sulfation, phosphorylation, grafting, and interpenetrating polymer network (IPN) formation, with their effects on structural characteristics, physicochemical properties, biointerfacial interactions, biological functions, and pharmaceutical performance. Particular emphasis is placed on identifying structure–property–biofunction relationships and on extracting design principles that can guide the selection and combination of reengineering strategies for specific applications. Reengineering of natural polysaccharides provides a versatile strategy to overcome the inherent limitations of native materials and to develop polysaccharide-based platforms with tailored physicochemical and biological properties. Different modification approaches offer distinct functional advantages: carboxymethylation can improve hydrophilicity and pH-responsive swelling, whereas sulfation and phosphorylation introduce anionic functionalities that modulate charge-dependent interactions and biological responses. Similarly, grafting and interpenetrating polymer network (IPN) formation provide greater control over mechanical strength, swelling, stability, drug-loading capacity, and controlled-release behavior. Collectively, these findings demonstrate that the performance of reengineered polysaccharides is determined not by the modification strategy alone, but by the combined effects of the resulting molecular structure, physicochemical characteristics, and interactions with biological environments. Despite these advances, the development of predictable structure-function relationships remains challenging because of variations in polysaccharide source, molecular weight, degree and distribution of modification, and characterization methodologies. Therefore, future research should shift from empirical modification toward rational, function-driven polysaccharide reengineering, in which structural parameters are deliberately controlled to achieve predefined material and biological outcomes.

## 1. Introduction

Over time, there has been a growth in the demand for safer and more effective products for pharmaceutical and medical purposes. As a result, it is now considered essential to explore new technologies for enhancing drug delivery and developing better medical applications. In particular, polymeric systems are of interest for a variety of medicinal applications, including drug delivery. The pharmaceutical and medical industries make extensive use of polymers due to their extraordinary and noteworthy properties, such as cost-effectiveness, durability, stability, biocompatibility, efficiency, and biodegradability [1].

Extensive research has been done on both synthetic and natural polymers. Most synthetic polymers have some drawbacks, like toxicity, high cost, side effects, non-renewable sources, and poor patient compliance [2]. Natural polymers have been applied in a variety of drug delivery applications, including emulsification, controlled/sustained drug release, rheology modifiers, film coating, solubilization, gelling agents, bulking agents, and drug encapsulation, among others [3,4,5]. The increasing use of natural polymers in delivering drugs suggests the need for research and development into new natural polymers for eventual commercialization. The use of natural polysaccharides is one of them. They are used for novel drug delivery systems and for personalized medicines.

Polymers were traditionally regarded as pharmacologically inactive excipients, as they were not expected to exert intrinsic therapeutic effects or directly modify the pharmacological action of drugs. Nonetheless, it is now recognized that polymeric excipients can influence the rate and extent of drug absorption and, consequently, drug bioavailability [6]. In this sense, the modulation of drug release and absorption has been the subject of extensive investigation in recent years [7]. Despite the growing application of natural polysaccharides in pharmaceutical and biomedical systems, a significant research gap remains in understanding how commonly used modification strategies translate into predictable changes in physicochemical properties, drug-delivery performance, and therapeutic activity.

**Figure 1:**
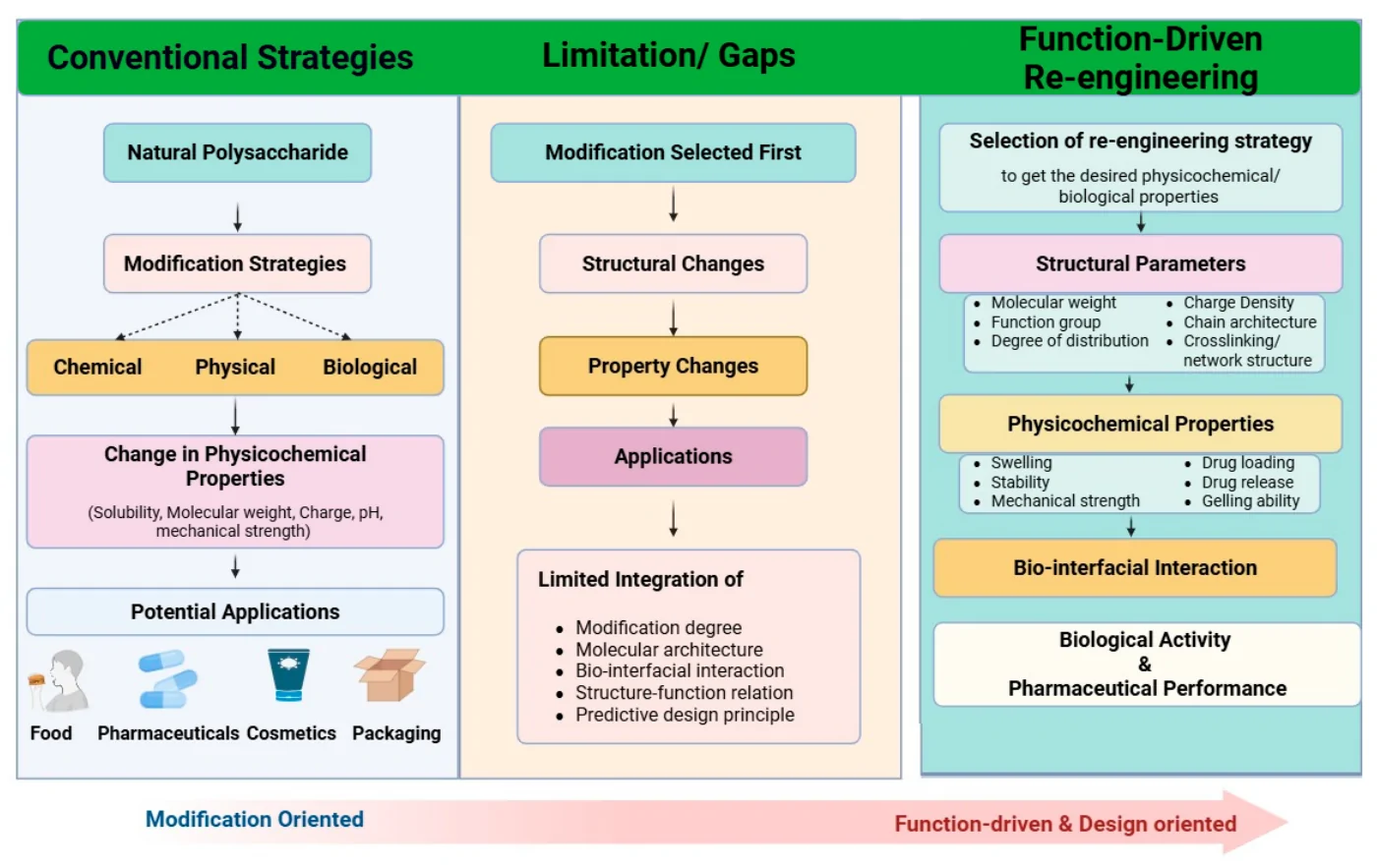
Comparative conceptual framework illustrating the transition from conventional modification-oriented strategies to function-driven polysaccharide re-engineering.

In this review, we use the term polysaccharide reengineering to describe the redesign of natural polysaccharides through controlled chemical, physical, or network-level modifications to establish clear relationships between their structure, physicochemical properties, biointerfacial interactions, and biological functions. Accordingly, modification represents the individual structural or chemical operation, whereas reengineering refers to the rational selection and, where appropriate, combination of such modifications to achieve a predefined functional objective. Existing reviews have mainly described individual modification techniques or summarized their applications, while comparatively less attention has been given to connecting the resulting physicochemical and biological outcomes from different modification techniques. Unlike previous reviews that primarily classify polysaccharide modification strategies and summarize their effects and applications, this review examines polysaccharide reengineering as a structure-driven approach, emphasizing how specific molecular modifications translate into changes in physicochemical properties and biofunctional outcomes. Figure 1 illustrates the conceptual difference between conventional polysaccharide modification strategies and the function-driven re-engineering approach discussed in this review. It highlights how the present approach connects structural changes with physicochemical properties, bio-interfacial interactions, and pharmaceutical performance. Particular emphasis is placed on carboxymethylation, sulfation, phosphorylation, grafting, and interpenetrating polymer network (IPN) formation, as these approaches represent widely investigated and functionally important strategies for tailoring the molecular structure, physicochemical, and biological properties of the polysaccharides. These modification methods were selected because they introduce distinct chemical functionalities or polymeric architectures and consequently provide different mechanisms for regulating properties such as solubility, charge, swelling, stability, mechanical characteristics, and drug-release behavior. Particular emphasis is placed on understanding the relationship between the type and extent of modification and the resulting functional and therapeutic properties, thereby highlighting the potential of structurally engineered polysaccharides for advanced biomedical and pharmaceutical applications [7].

## 2. Methodology

A systematic literature search was conducted using PRISMA 2020 guidelines [8]. The search was performed in three major electronic databases: PubMed, Scopus, and Web of Science. The search covered publications from 2000 to 2026. The search terms were organized into three main concepts: (i) polysaccharides, (ii) chemical or physicochemical modification, and (iii) functional, biological, pharmaceutical, or drug-delivery applications.

*Studies were considered eligible when they met the following inclusion criteria:* (i) published between 2000 and 2026; (ii) investigated the modification of a polysaccharide; (iii) reported sufficient information regarding the modification procedure and/or resulting material properties; (iv) provided evidence relevant to the functional, biological, pharmaceutical, or drug-delivery properties of the modified polysaccharide; and (v) written in English.

*Studies were excluded when they:* (i) did not involve polysaccharides; (ii) did not investigate polysaccharide modification; (iii) focused exclusively on the extraction, isolation, purification, or characterization of unmodified polysaccharides without evaluating modification; (iv) did not report relevant functional, biological, pharmaceutical, or drug-delivery outcomes; (v) were conference abstracts, editorials, letters, corrections, theses, dissertations; (vi) were duplicate publications; or (vii) were not available in English.

Table 1 shows the search string that was used to search in each database. The search resulted in a total of 5686 papers, of which 1806 were obtained from Scopus, 2682 were obtained from PubMed, and 1198 were obtained from Web of Science.

*Study Selection:* Before screening, duplicate records (*n* = 3426) and irrelevant records (*n* = 1230) were removed. Subsequently, 1030 papers underwent title and abstract screening, during which 891 papers were excluded based on the predefined exclusion criteria. This left 139 full-text articles for eligibility assessment.

## 3. Natural Polysaccharides

Natural polymers are preferred over synthetic ones because they are more readily available, amenable to chemical modification, renewable, affordable, nontoxic, stable, hydrophilic, biocompatible, and biodegradable than expensive synthetic polymers that have long-standing synthetic methods and environmental and toxicity issues [3]. The most common natural biopolymers are polysaccharides, which are becoming more popular and useful materials in a variety of biomedical sectors because of their inherently remarkable and desirable characteristics. A polysaccharide’s backbone is made up of monosaccharide units, which are typically linked together by an ether bond, an O-glycosidic linkage [9]. When hydrolyzed, these polysaccharides produce simple sugars including glucose, galactose, mannose, arabinose, xylose, uronic acids, etc. [10,11]. Although natural polysaccharides are generally known for their positive safety characteristics, it is necessary to emphasize that attributes like biodegradability, biocompatibility, and non-toxicity do not necessarily apply universally to all members of this category.

The pharmaceutical performance of natural polysaccharides is strongly dependent on their molecular architecture and chemical composition. Unlike synthetic polymers with relatively well-defined structures, natural polysaccharides exhibit considerable structural diversity arising from differences in molecular weight, monosaccharide composition, glycosidic linkages, degree of branching, and the distribution of functional groups. These structural characteristics determine their hydration, intermolecular interactions, viscosity, swelling, gelation, degradation, and biological recognition, and consequently influence their suitability for pharmaceutical and biomedical applications [12]. Importantly, the native molecular architecture also determines the response of a polysaccharide to chemical modification. Previous studies have shown that chemical modification can alter molecular weight and other structural characteristics, which can subsequently enhance or modify biological activity. High-molecular-weight chitosan (>300 kDa) is less soluble compared with its oligomer counterparts, while being associated with a higher potential for inflammation induction after implantation [10]. The heat resistance and degradability of the starch-containing structures depend heavily on the composition of amylose and amylopectin in relation to crystallinity [13]. Alginate’s gelling capability and lack of immunogenicity strongly correlate with G/M ratios that vary from species to species [13].

### 3.1. Sources of Natural Polysaccharides

**Figure 2:**
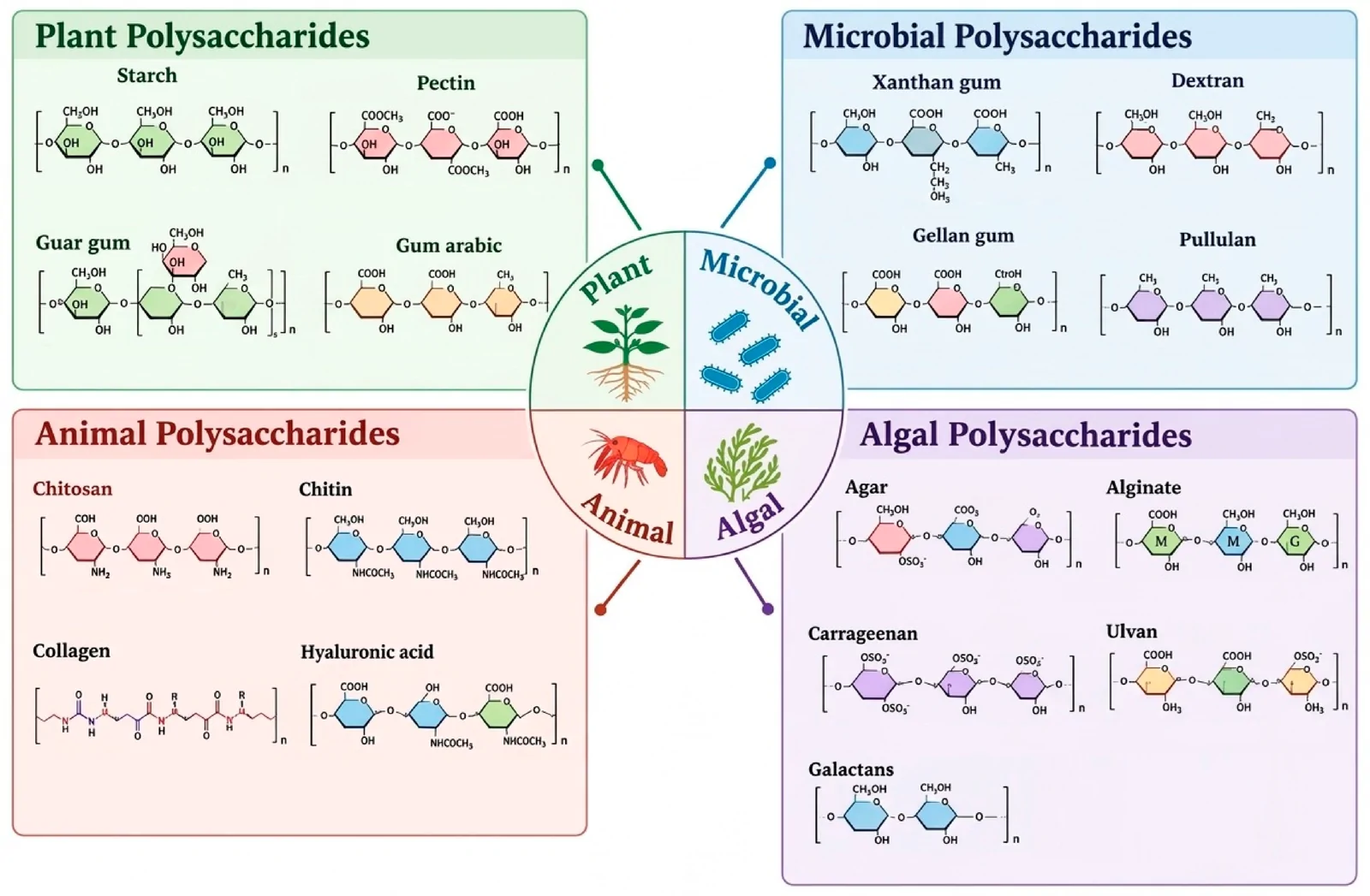
Natural polysaccharides from different sources.

Based on where they come from, these naturally occurring polysaccharides are separated into four groups: microbial, algal, animal, and plant polysaccharides (Figure 2). Table 2 shows some important natural polysaccharides, their source, and pharmaceutical applications.

### 3.2. Functionality and Application

The usefulness of native polysaccharides is frequently restricted by their inherent physicochemical characteristics. These polysaccharides can be tailored or modified to improve their functionality and fit particular applications. Purposes of tailoring natural polysaccharides are:

- Site-specific targeting: Modification with ligands (folate, mannose, galactose) can promote tumor- or tissue-specific uptake through receptor-mediated transport, increasing efficacy and reducing toxicity [10].
- Temperature and humidity stability: Cross-linking decreases water solubility and protects against heat-induced decomposition, thus prolonging the shelf life and facilitating use in processes like hot-melt extrusion and spray drying [10].
- Alterations in solubility: Incorporating hydrophilic groups (carboxymethyl, sulfate, phosphates) increases the solubility of otherwise hydrophobic polysaccharides (cellulose and chitin), expanding processing opportunities [13].
- Touch sensitivity: Softening through plasticizing and graft copolymerization with flexible polymer chains increases touch sensitivity and transparency, making it possible to produce wound dressings and flexible sensors as well as edible films.
- Hemocompatibility: Modification by PEGylation or introduction of zwitterionic groups lowers protein adhesion and improves blood and mucosal tolerance [14].
- Timed biodegradation: The degree of crosslinking determines the ease with which enzymes can break down the material, offering a mechanism for programmed degradation kinetics.
- Low toxicity: Partial deacetylation, purification, or separation of high- and low molecular weight fractions, or removal of endotoxins, ensures that the modified polymers meet safety standards for injection or mucosal delivery.

## 4. Tailoring of Natural Polysaccharides

Structural reengineering of natural polysaccharides involves the application of chemical and physical modification strategies to tailor their physicochemical properties and improve their functional performance. Various structural modification approaches and their effects on the physicochemical properties of natural polysaccharides are discussed below.

### 4.1. Chemical Modification

#### 4.1.1. Carboxymethylation

**Figure 3:**
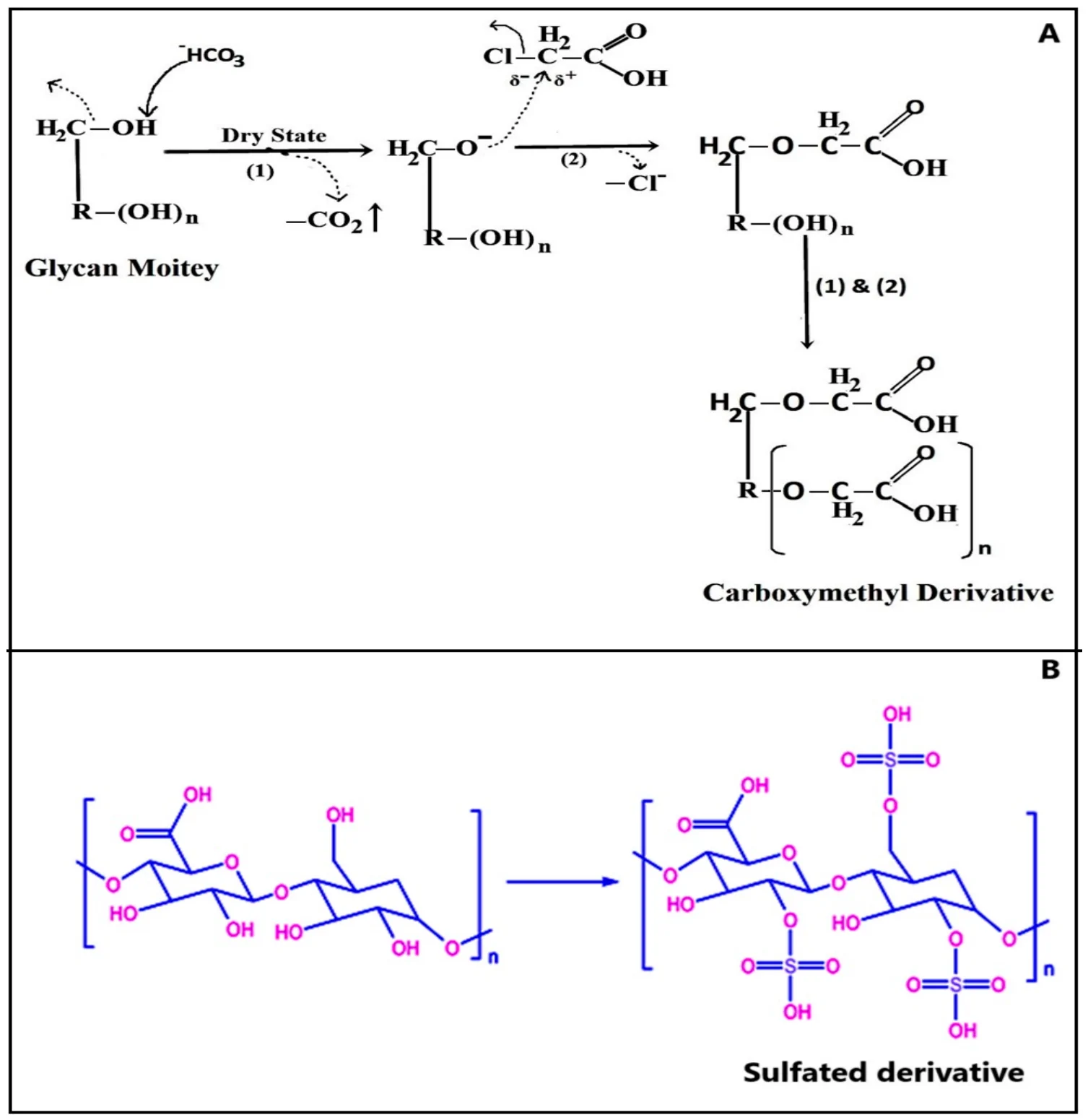
(**A**) Carboxymethyl modification of polysaccharide; (**B**) sulfation modification of polysaccharide.

Among various chemical modification processes, carboxymethylation has been widely studied due to its simplicity and efficiency. Carboxymethylation is the introduction of carboxymethyl groups to the polysaccharide chain, increasing the number of hydrophilic and ionizable functional groups within the polymer structure (Figure 3A) [15]. These days, carboxymethylation is utilized to alter the physical, chemical, and structural features of natural polysaccharides in order to broaden their range of applications. This modification generally enhances water solubility, water-holding capacity, and swelling behavior because of increased polymer–water interactions [16]. Cellulose is an example of such polysaccharides, where cellulose becomes soluble on account of its conversion into carboxymethyl cellulose (CMC) by virtue of disrupting the hydrogen bonding present in natural cellulose by means of carboxymethyl groups [13]. The same applies to other refractory polysaccharides like chitin, where the carboxymethylation results in the production of water-soluble products with broader ranges of pH as compared with natural chitosan [10]. Solubility is directly correlated with the degree of substitution, wherein the presence of higher numbers of carboxymethyl groups creates stronger electrostatic forces that minimize interchain aggregation [17,18]. One study reported that increasing the degree of substitution (DS) of the carboxymethylated polymer resulted in enhanced water solubility and an increase in its average molecular mass. This effect can be attributed to the incorporation of carboxymethyl groups into the polymer backbone, which increases the number of hydrophilic and ionizable groups within the polymer structure. Consequently, the increased ionic character and molecular mass can alter the polymer–water interactions and, thereby, improve its aqueous solubility. Since molecular mass and water solubility are important physicochemical properties that can influence the biological activity, bioavailability, and functional performance of polymers, changes in DS may consequently affect their biological properties [19].

Carboxymethylation introduces ionizable carboxylic groups (–COOH) into the polymer network, imparting pH-responsive behavior. In acidic environments, these groups remain predominantly protonated, resulting in relatively weak electrostatic repulsion between adjacent polymer chains and consequently limited network expansion. As the pH increases, the carboxylic groups progressively deprotonate to form negatively charged carboxylate groups (–COO⁻), increasing electrostatic repulsion within the polymer network. This repulsive force promotes expansion of the polymer chains and increases the uptake of water, thereby enhancing the swelling ability of modified polysaccharide [20,21]. The food industry uses tamarind seed polysaccharide (TSP) as a gelling, stabilizing, and thickening agent. Although TSP is a water-soluble polysaccharide, it has undergone chemical modifications such as carboxymethylation due to its drawbacks, including an unpleasant odour, a dull colour, and a rapid rate of degradation [22]. TSP’s carboxymethylation gives the polymer its anionic character [23]. The carboxymethyl tamarind kernel polysaccharide (CMTSP) has a longer shelf life because carboxymethylation increases its viscosity and decreases its biodegradability. Due to this, CMTSP has a lot of potential as a novel drug carrier in controlled-release formulations [23]. In a recent work, Kaur et al. formulated and evaluated tropicamide-loaded CMTKP nanoparticles for ocular delivery [24]. Ionotropic gelation was used to create the CMTKP loaded with tropicamide. Results suggest that the ex vivo ocular penetration of tropicamide by the nanoparticulate formulation was equivalent to that of its aqueous solution [24].

In another study, Kaity S. et al. developed a sodium carboxymethyl ether of locust bean gum, a naturally occurring polysaccharide, to maximize its capacity for attracting water and to boost its aqueous solubility [25]. Toxicity and biodegradability studies of carboxymethyl locust bean gum revealed that it is safe for internal use. The interpenetrating polymer network microspheres of buflomedil hydrochloride were prepared by using carboxymethyl locust bean gum and poly(vinyl alcohol) for controlled drug delivery [25].

Collectively, these findings indicate that the degree and distribution of carboxymethyl substitution are not merely chemical descriptors, but key structural parameters governing charge density, hydration, solubility, and subsequent biological interactions.

#### 4.1.2. Sulfation

Sulfated polysaccharides have emerged as a major focus in polysaccharide research in recent years due to their enhanced biological activities. Among the various chemical modification strategies, sulfation is particularly notable for its ability to significantly improve polysaccharide bioactivity. Sulfation involves the introduction of sulfate groups (–OSO₂OH) into the polysaccharide backbone (Figure 3B) [26]. Following sulfation, the hydroxyl groups undergo substitution, leading to changes in steric hindrance and electrostatic repulsion. In polysaccharide chains, hydroxyl groups serve as the primary substitution sites for sulfation. Sulfate groups are generally introduced by replacing the hydroxyl groups at specific carbon sites (C-2, C-4, or C-6) of the polysaccharide backbone through an esterification process. The incorporation of sulfate groups can also introduce additional reactive sites along the polysaccharide backbone, potentially enhancing its chemical reactivity [27].

The introduction of sulfate groups converts the relatively neutral polysaccharide into a polyanionic polymer, thereby changing its charge density, molecular conformation, solubility, and biological properties [28]. Sulfation generally increases the hydrophilicity and water solubility of polysaccharides because sulfate groups are strongly polar and ionizable in aqueous media. The negatively charged sulfate groups promote interaction with water molecules and can increase hydration of the polymer chains [29]. The incorporation of bulky and negatively charged sulfate groups can alter the three-dimensional conformation and intermolecular interactions of polysaccharide chains. Sulfation may affect hydrogen bonding, chain flexibility, molecular aggregation, and interactions with ions and proteins. Consequently, properties such as viscosity, rheological behavior, swelling, and hydration can be modified. The extent of these changes depends strongly on the degree and position of sulfate substitution [28].

High-bioactivity sulfated polysaccharides can be achieved only through appropriately controlled sulfation techniques, as the degree and pattern of sulfate group substitution play a critical role in determining their functional enhancement [26]. This modification is typically carried out through chemical reactions using sulfating agents such as sulfur trioxide–pyridine complexes, chlorosulfonic acid, or sulfuric acid [26].

##### Sulfation Through Chlorosulfonic Acid–Pyridine

Among various sulfation methods, the chlorosulfonic acid–pyridine method stands out for its efficiency, control, and reproducibility. The sulfation of natural polysaccharides using the chlorosulfonic acid–pyridine method involves a series of well-defined steps. Initially, the polysaccharide is dissolved in an anhydrous organic solvent like dimethylformamide or dimethyl sulfoxide to ensure a homogeneous solution. Chlorosulfonic acid is then carefully combined with pyridine to form a reactive pyridinium-chlorosulfate complex [28]. This complex is gradually added to the polysaccharide solution under constant stirring, maintaining a controlled temperature to ensure efficient sulfation and minimize polysaccharide degradation. The degree of sulfation is influenced by the molar ratio of chlorosulfonic acid to polysaccharide, reaction temperature, and duration. After achieving the desired degree of sulfation, the reaction is neutralized using a base like sodium bicarbonate to stop the reaction and remove excess reagents [26]. The sulfated polysaccharide is precipitated by adding the reaction mixture to a nonsolvent such as ethanol or acetone. Finally, the product is washed multiple times with ethanol or acetone to eliminate residual reagents and byproducts, then dried under vacuum or in a low-temperature oven to obtain the final sulfated polysaccharide [30,31]. This method efficiently and controllably introduces sulfate groups into various polysaccharides, enhancing their bioactivity for diverse drug delivery applications. In a study, polysaccharides from *Medicago sativa* L. stems were successfully sulfated using the chlorosulfonic acid–pyridine method under three distinct reaction conditions, resulting in sulfation derivatives (SAPS-1, SAPS-2, and SAPS-3). The spectroscopic analysis confirmed effective sulfation of the polysaccharide, with molecular weights of SAPSs decreasing as the degrees of substitution increased (ranging from 0.10 to 0.23) [32].

##### Sulfation Through Sulfur Trioxide–Pyridine Complex

Among various sulfation methods, the sulfur trioxide–pyridine complex stands out for its efficiency and specificity. This method is particularly valuable for its precise control over the degree of sulfation, making it highly suitable for pharmaceutical and biomedical applications. The sulfur trioxide–pyridine method is a widely used approach due to its ability to produce highly sulfated polysaccharides with consistent quality [33]. In this method, polysaccharides are dissolved or suspended in an anhydrous organic solvent to create a homogeneous solution. Sulfur trioxide (SO₃) is then complexed with pyridine (C₅H₅N) to form a highly reactive sulfur trioxide–pyridine complex, which serves as a potent sulfating agent. This complex is gradually added to the polysaccharide solution under constant stirring, with the reaction typically conducted at a controlled temperature to ensure efficient sulfation and minimize degradation of the polysaccharide. Once the desired degree of sulfation is achieved, the reaction mixture is neutralized with a base like sodium bicarbonate, halting the reaction and removing excess reagents [33]. The sulfated polysaccharide is then precipitated by adding the mixture to a nonsolvent such as ethanol or acetone, followed by multiple washes to eliminate residual reactants and byproducts. Finally, the product is dried under a vacuum or in a low-temperature oven to obtain the sulfated polysaccharide. Chen et al. recently used the sulfur trioxide–pyridine complexation approach to modify the rice bran polysaccharide. Investigations were conducted into how sulfation modification affected the polysaccharide’s structure, bacterial inhibition, physicochemical characteristics, and inhibitory mechanism. The findings indicated that sulfation changed the fraction of monosaccharides in rice bran polysaccharides, raised the molecular weight, and decreased the amount of protein and carbohydrates. A successful modification was confirmed by UV, FT-IR, and NMR spectra, with sulfation mostly taking place on hydroxyl groups at C-6 [34].

##### Sulfation Through Concentrated Sulfuric Acid

Concentrated sulfuric acid and pyridine were combined in the correct proportions to create an esterification reagent, which was then stirred with n-butanol in an ice-water bath [35]. Ammonium sulfate was added to the reaction mixture, followed by the slow addition of polysaccharide powder after thorough mixing. The reaction proceeded at room temperature for 3 h, with the pH maintained at 7 using an ice-cold sodium hydroxide solution. Ethanol was used to precipitate the sulfated polysaccharides, which were collected via centrifugation, dialyzed, and freeze-dried to obtain the final sulfated polysaccharide product [28,35].

Overall, the introduction of sulfate groups into polysaccharide chains establishes a structure–property–biofunction relationship that governs the functional performance of sulfated derivatives. These structural changes can modify physicochemical properties such as water solubility, hydrophilicity, molecular weight, viscosity, and aggregation behavior. The resulting changes in charge distribution and chain conformation may enhance interactions with proteins, cell-surface receptors, and other biological targets, potentially improving antioxidant, antimicrobial, anti-inflammatory, anticoagulant, and other biological activities. However, the biofunctional effects of sulfation are strongly dependent on the degree of substitution, substitution pattern, molecular weight, and structural characteristics of the parent polysaccharide.

#### 4.1.3. Phosphorylation

The process of phosphorylating natural polysaccharides entails the addition of phosphate groups to the polysaccharide structures, thereby improving their functional characteristics and suitability for a range of applications [36]. The hydroxyl group of the polysaccharide chains typically serves as the site for substitution. The availability of naturally occurring phosphorylated polysaccharides is restricted; consequently, in recent years, the phosphorylation of polysaccharides has emerged as a crucial method for their structural modification [37]. The modification changes the chemical environment of the polymer by introducing ionizable phosphate groups. Consequently, the polysaccharide acquires a higher negative charge density, and its molecular conformation and intermolecular interactions can change. The extent of these changes depends on the degree of substitution, reaction conditions, polysaccharide structure, and position of phosphorylation [38]. Phosphorylation generally improves the water solubility of polysaccharides. The phosphate groups are hydrophilic and can become negatively charged in aqueous media, increasing the interaction between the modified polymer and water. This is particularly useful for polysaccharides that originally have poor water solubility [38]. Phosphate modification can increase the swelling capacity and water uptake of polysaccharides because the introduced phosphate groups have a strong affinity for water. The negatively charged groups can also promote expansion of polymer chains through electrostatic repulsion. In starch systems, phosphate content is associated with changes in swelling power and other hydration-related properties. However, the magnitude and direction of the effect depend on the degree of phosphorylation and the structure of the original polysaccharide [39]. Phosphate modification can alter the thermal stability, gelatinization, and thermal transition behavior of polysaccharides. This occurs because phosphate groups modify hydrogen bonding, molecular interactions, and crystalline/amorphous organization within the polymer [39].

Phosphorus oxychloride, phosphate, phosphoric acid and its anhydride, and phosphorus pentoxide are the methods for phosphorylation that are most frequently utilized [38].

##### Phosphoric Acid and Its Anhydride Method

**Figure 4:**
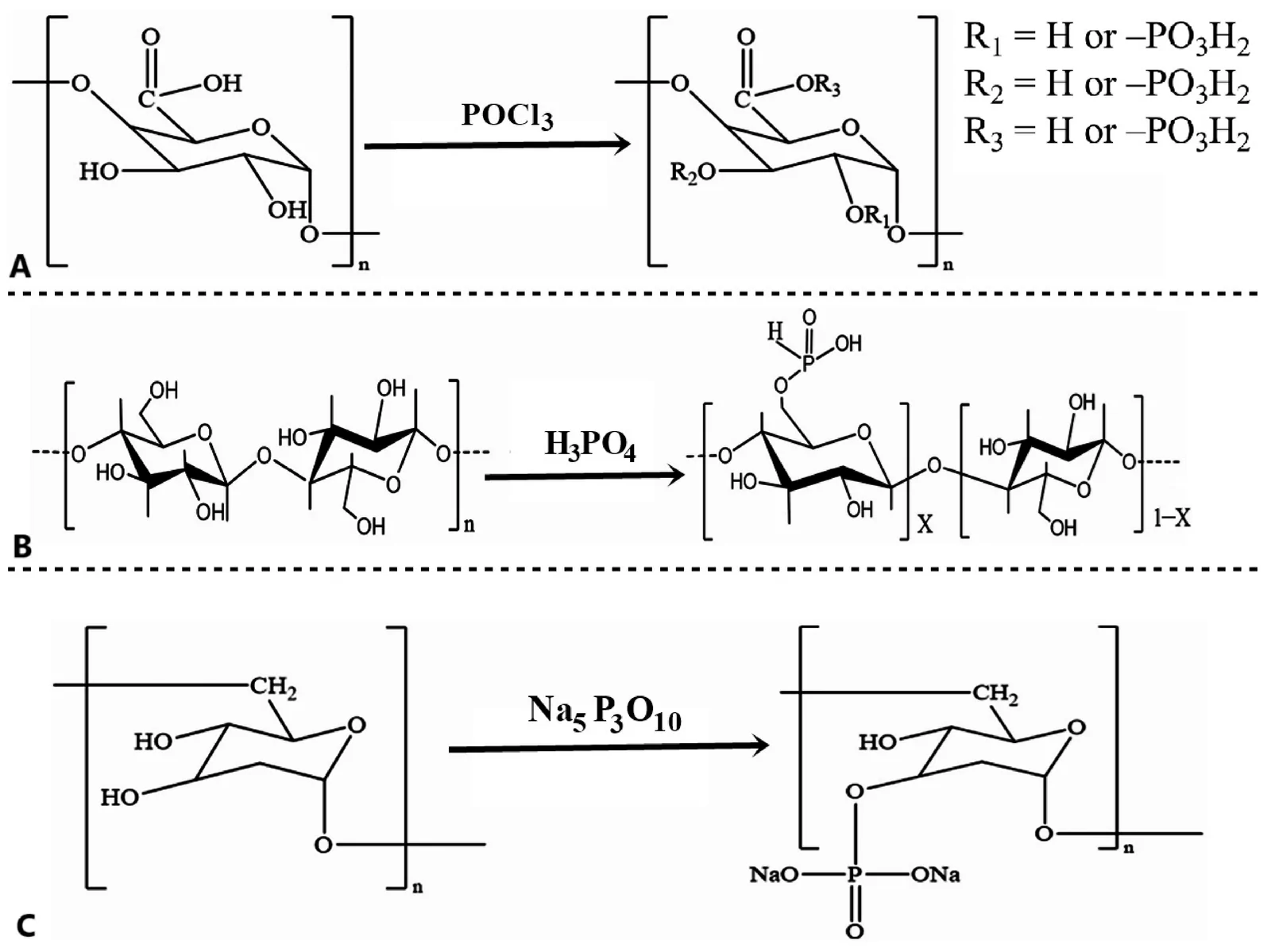
Phosphorylation of polysaccharides through: (**A**) Phosphorus oxychloride modification method. (**B**) Phosphoric acid and its anhydride modification method. (**C**) Sodium tripolyphosphate modification method.

Using this approach, urea and dimethyl sulfoxide are first used to dissolve the powdered polysaccharide. After adding phosphoric acid to the mixture, the reaction is allowed to run for six hours at 100 °C [40]. Following the procedure, the final phosphorylated polysaccharide is obtained by lyophilizing the product, which has been dialyzed to eliminate contaminants. Benefits of this method include minimal cost, short operating stages, and interoperability with other instruments. Limited current uses can arise from polysaccharide breakdown and reduced yield of the intended product due to the strong heat created during the phosphoric acid reaction (Figure 4B).

##### Phosphoryl Chloride Method

The phosphoryl chloride method, or phosphorus oxychloride method, is a synthetic approach that yields highly substituted phosphorylated compounds with a high degree of substitution (DS) and with rapid reaction durations. Using an ice-water bath to regulate the reaction temperature and time, phosphorus oxychloride (POCl₃) and pyridine are gradually added after the powdered polysaccharide is dissolved in dimethylformamide [41]. Following the process, centrifugation, freeze-drying, and alcohol precipitation are used to recover the phosphorylated polysaccharides. This approach, however widely used, has many disadvantages, such as a strong reaction process, very hazardous byproducts, and the production of unpleasant fumes [42] (Figure 4A).

##### Phosphate Method

Because the phosphate technique has the advantage of not readily breaking down polysaccharides, it is frequently used for phosphorylation modification. Despite this advantage, there are also significant disadvantages to the technique, including the low polysaccharide reaction activity, which can lead to comparatively poor degrees of substitution and yield. Sodium trimetaphosphate (STMP) and sodium tripolyphosphate (STPP) are common phosphates employed in this approach [43] (Figure 4C). Usually, distilled water is used to dissolve polysaccharide powder, and then a 6:1 ratio of STPP and STMP is added in accordance with optimal phosphorylation conditions. At 80 °C, the reaction is conducted for six hours. Following the reaction, neutralization, dialysis, and freeze-drying are used to extract the product [44].

Overall, the degree and position of phosphate substitution determine the charge density, molecular conformation, and intermolecular interactions of the modified polymer, which in turn influence its physicochemical and biological properties. The incorporation of hydrophilic and ionizable phosphate groups generally enhances water solubility, swelling, water uptake, and hydration, while also modifying hydrogen bonding, thermal behavior, gelatinization, and the organization of crystalline and amorphous regions. These structural and physicochemical changes can affect interactions with proteins, ions, and biological interfaces, potentially improving functional properties such as antioxidant, antimicrobial, anti-inflammatory, and other bioactivities.

### 4.2. Grafting

**Figure 5:**
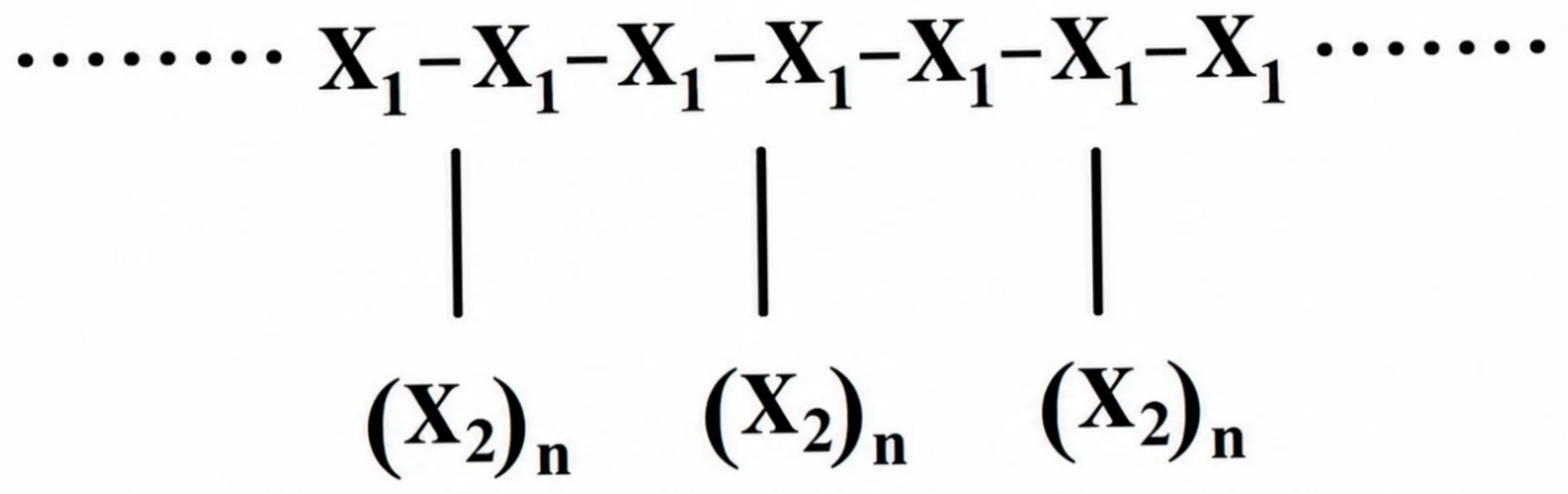
The generalized structure of a graft copolymer.

Recently, grafted polysaccharides have attracted increasing attention as a promising strategy for the development of effective drug delivery systems [45]. It is an important method for adding advanced characteristics to backbone polymers without changing their basic function. Graft copolymerization gives researchers a technique to add specific characteristics to backbones for specialized purposes without impacting their biodegradability [46]. Grafting is widely recognized as a versatile approach for combining polysaccharides with other polymers or porous materials to fabricate stimuli-responsive and targeted delivery systems, particularly for cancer therapy [47]. In grafting, the monomers are joined on the surface of the polysaccharide or polymer backbone at several points [48]. Numerous researchers have successfully grafted onto various polysaccharide backbones using different vinyl monomers, a variety of initiators, oxidizing agents, binary vinyl monomeric mixtures, and radiation techniques [49]. The generalized structure of a graft copolymer, which incorporates a monomer (X2) on the backbone of a polymer with a building unit (X1), is given below in Figure 5 [50]. The fundamental idea behind graft copolymerization synthesis is the formation of active sites on the backbone, which can take the shape of free radicals or a functional group. When monomers are polymerized onto active sites, graft copolymerization takes place.

#### 4.2.1. Chemical Methods

Chemical approaches are those that use chemicals to initiate the active sites on the polymer backbone. Chemical grafting has employed a variety of redox initiators, including Lewis acids, metal carbonyls, and strong bases [51]. Vinyl monomers have been grafted onto polymeric backbones using a range of redox systems and free radical initiators, such as Fenton’s reagent, dibenzoyl peroxide, azobis(isobutyronitrile), ceric ammonium nitrate, potassium persulphate, and potassium permanganate. In a study, Chunhua et al. prepared chitosan-graft-poly(ɛ-caprolactone) amphiphilic copolymer micelles for the delivery of 5-fluorouracil [52]. Ring-opening polymerization was used to generate the grafted polymer, which was then examined using FTIR and ^1^H NMR spectroscopy. The findings showed that 5-fluorouracil’s release was under control and that its half-time could last up to 54.46 h, which is significantly longer than that of free 5-fluorouracil. Furthermore, 5-fluorouracil-loaded micelles may exhibit similar cytotoxicity against A549 cells and postpone the drug’s release.

#### 4.2.2. Radiation-Induced Grafting

When grafting of polymers is done by using radiation as an initiating system, it is called radiation-induced graft copolymerization. The energy from the radiation is deposited in the substance that needs to be modified. Highly reactive free radicals will be produced from this energy, which will lead to secondary reactions with molecules already present in the medium. These free radicals quickly interact with the proper functional monomers to establish covalent connections, which in turn promote the formation of macromolecular chains without the need for chemical initiators [53]. By choosing the right irradiation and reaction circumstances, the degree of grafting in the copolymer can be modified to create precisely designed selective copolymers for certain applications. Either low-energy radiation or high-energy radiation can be used to graft polysaccharides.

##### Low-Energy Radiation-Induced Grafting

Free radicals are generated on the backbone as a result of homolytic fission, which happens when the backbone is subjected to low-energy radiation like UV and visible light. Grafting can happen in the presence of low-energy radiation with or without a photoinitiator [54].

##### High-Energy Radiation-Induced Grafting

Graft copolymerization of a variety of backbones has been accomplished using high-energy radiation such as X-rays, gamma rays, or accelerated electrons with energies ranging between 3.0 MeV and 6.5 MeV. The most often used radiation is gamma rays, and Co-60 (with a half-life of 5.3 years) is the most common radiation source of gamma rays [55].

#### 4.2.3. Microwave Radiation-Induced Grafting

Another significant energy source that is becoming an efficient tool for graft copolymerization is microwave radiation. The rapid and homogeneous core processing of reactants is a benefit of microwave radiation. Microwave radiation can quickly transmit energy into the bulk of the reaction mixture, which causes the materials in the reaction mixture to interact with one another quickly [56,57,58]. For practically all of the grafting processes of relevance here, microwave irradiation considerably lowers the need for unsafe and toxic solvents and speeds up the reaction time, resulting in high production yield and product selectivity. Compared with their traditionally synthesized counterparts, microwave-produced polysaccharide copolymers demonstrate improved characteristics for commercial exploitation. The potential use of the modified moringa polysaccharide in hydrogel production for drug delivery applications was investigated by Singh et al. The radiation-induced graft-copolymerization of N-vinylimidazole onto the moringa polysaccharide was used to create the polysaccharide-based hydrogels [59]. Levofloxacin was shown to release slowly from the hydrogels without experiencing any burst effects. The authors reported that the polymers can be employed for gastrointestinal drug delivery systems. In another study, Iqbal et al. synthesized hydroxypropyl methylcellulose-graft-(polyvinylalcohol-co-acrylic acid) hydrogels by microwave-induced irradiation [60]. The authors reported that in comparison with the traditional hydrothermal method, the microwave-induced irradiation method significantly reduces the reaction time needed for polymer crosslinking from several hours to a few minutes. The consistent porosity, increased swelling ratio, and high drug encapsulation were found as a result of the rapid and instantaneous penetration of microwave energy across the surface. Results show that the controlled delivery of captopril over a longer time period was made possible by microwave radiation-induced hydrogel synthesis, which also produced a suitable drug carrier. It could be a good candidate to demonstrate its value in the treatment of hypertension and a few other cardiac conditions.

#### 4.2.4. Enzymatic Grafting

Enzymatic bioprocesses have been investigated as desirable alternatives to hazardous and non-specific chemical methods due to the decrease in input energy and environmental effect, as well as due to high specificity and selectivity features [61]. In contrast to chemical methods, which require harsh conditions and highly reactive substances, enzymatic reactions can be carried out under mild conditions and are less likely to result in undesirable byproducts. Commonly used enzymes for polymer modification purposes are laccases, tyrosinases, peroxidases, lipases, and transglutaminases [62].

Overall, grafting is a versatile and effective strategy for modifying polysaccharides to improve their functional properties and expand their applications in drug delivery systems. Various approaches, including chemical, radiation-induced, microwave-assisted, and enzymatic grafting, provide flexibility in reengineering the structure and properties of grafted polymers according to specific physicochemical and therapeutic requirements. Chemical and radiation-based methods offer efficient graft formation, whereas microwave irradiation can reduce reaction time and solvent consumption. Enzymatic grafting represents a more environmentally friendly alternative because it operates under relatively mild conditions with greater specificity and reduced formation of undesirable byproducts. Collectively, these grafting techniques have demonstrated considerable potential for developing reengineered polysaccharide-based advanced drug delivery platforms capable of achieving sustained and controlled drug release. Continued optimization of grafting conditions, biocompatibility, scalability, and reproducibility may further facilitate the translation of grafted polysaccharide systems from laboratory research to practical pharmaceutical and biomedical applications.

### 4.3. Interpenetrating Polymer Network (IPN)

**Figure 6:**
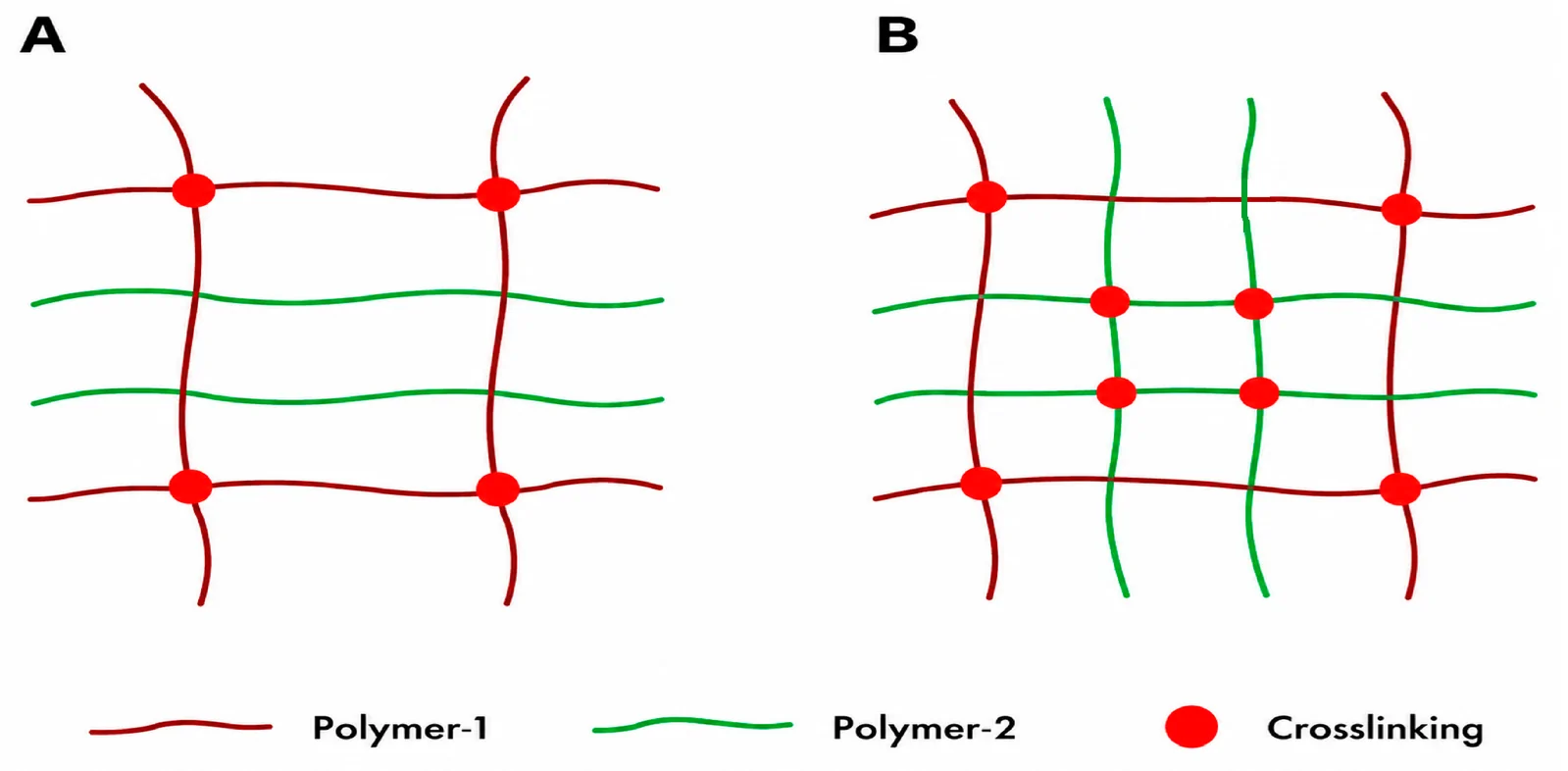
(**A**) Semi-IPN (only one polymer is crosslinked). (**B**) Full IPN (both polymers are crosslinked).

A pair of polymers combined in network form, at least one of which is synthesized and/or cross-linked in the near vicinity of the other, is referred to as an interpenetrating polymer network (IPN) (Figure 6) [63]. The primary function of IPN is to add novel properties to one of the components while keeping the essential qualities of the other component. Additionally, they differ from polymer complexes and graft copolymers that contain either chemical linkages or modest levels of cross-linking. IPN has several advantages because it combines the coactive properties of two polymers, such as the cross-linking or interpenetration of one natural polymer with another synthetic polymer. As a result, the resulting IPN exhibits better properties for the controlled release of drugs, has more pronounced phase stability, and has significantly improved mechanical properties at the final stage. Overall, reengineering through IPNs can retain the desirable characteristics of individual polymer components while providing enhanced mechanical strength, phase stability, swelling behavior, and drug-loading capacity. These structural advantages make IPNs particularly promising for controlled and sustained drug delivery, where the network architecture can be tailored to regulate drug diffusion and release.

The advantages and disadvantages of several common methods for polysaccharide modification and the research on the effect of polysaccharide modification on their physicochemical and biological properties are summarized in Table 3 and Table 4, respectively.

## 5. Biological Activity of Structurally Modified Natural Polysaccharides: Impact of Chemical Modification on Biological Activities of Polysaccharide

**Figure 7:**
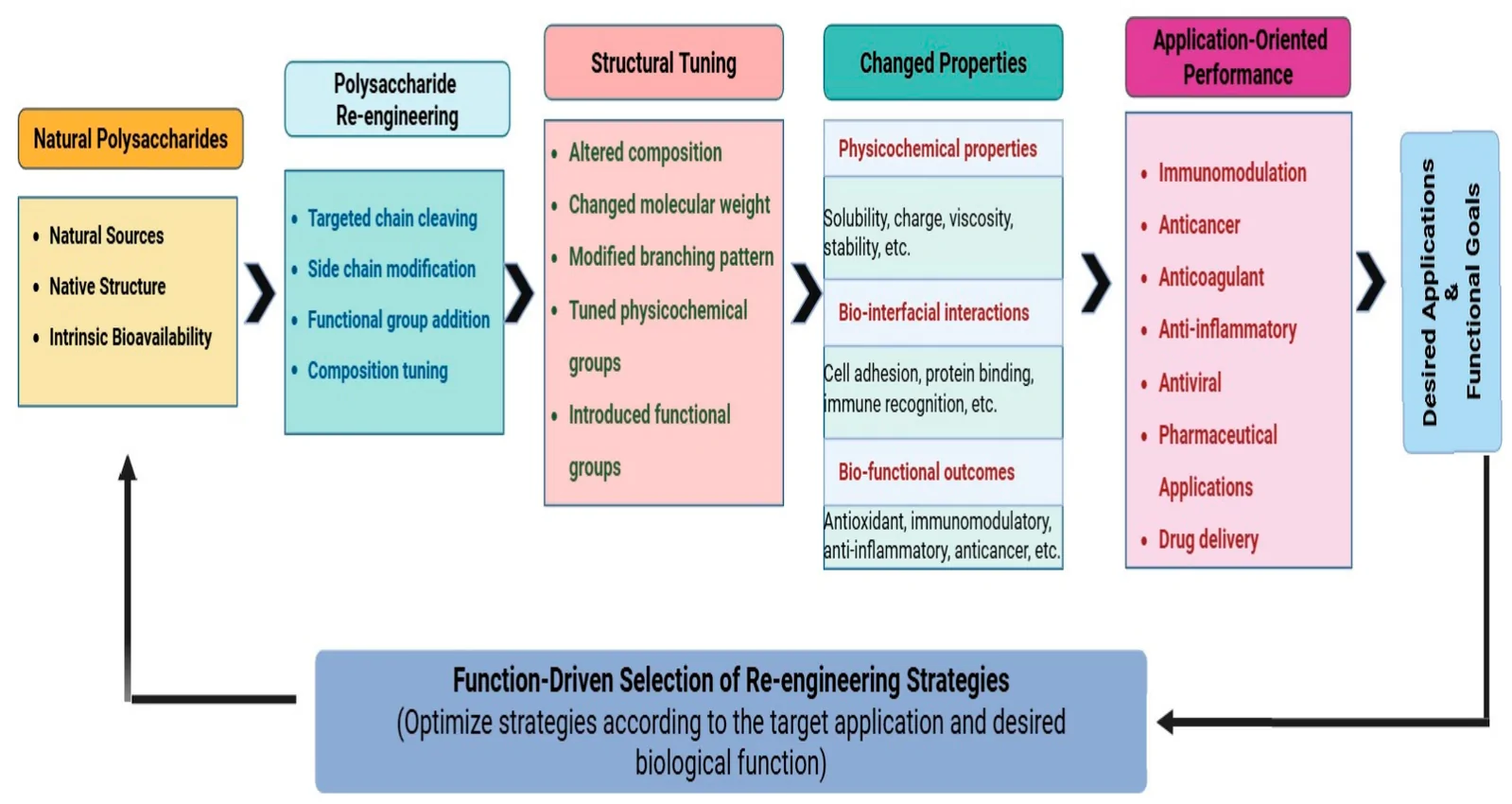
Schematic framework of polysaccharide reengineering strategies, property modifications, and application-oriented biofunctional outcomes.

Exploring the biological activity of structurally modified natural polysaccharides reveals their potential in various biomedical, therapeutic and food applications. Polysaccharides, derived from natural sources such as plants, seaweeds, and microbes, are inherently bioactive due to their complex structures and interactions with biological systems. Structural modifications can further enhance or alter these bioactivities, making them versatile materials for biomedical research, pharmaceuticals, food and cosmetics (Figure 7).

### 5.1. Antioxidant Properties

Free radicals are highly reactive molecules with unpaired electrons, generated as byproducts of normal cellular metabolism. Common examples include superoxide anion (O_2_−), hydroxyl radical (·OH), and nitric oxide (–NO). These reactive oxygen species (ROS) play dual roles in the body, participating in essential physiological processes but also posing potential risks when produced in excess, including chronic kidney disease, cancer, and cardiovascular disorders. Consequently, the search for natural antioxidants with low toxicity is of significant importance. Recent studies have highlighted the potential of chemically modified polysaccharides as effective antioxidants. These modified polysaccharides not only exhibit strong antioxidant properties but also have low toxicity and wide availability, making them potential candidates for pharmaceuticals, food, and cosmetic industries where antioxidant properties are crucial for preventing oxidative stress-related damage. Typically, the antioxidant activity of modified polysaccharide compounds surpasses that of their original forms. This enhanced antioxidant ability is likely due to a combination of multiple factors rather than a single cause. These factors include the degree of substitution, molecular weight, monosaccharide composition, content of modified groups (like sulfate, carboxymethyl group, among others), and the distribution and structure of these groups. Together, these elements contribute to the overall improvement in the antioxidant properties of the modified polysaccharides [82,83].

Studies have demonstrated that carboxymethylated polysaccharides exhibit superior antioxidant properties compared with their unmodified counterparts. The strong antioxidant activity of carboxymethylated polysaccharides likely involves the lone pair electrons on the carboxymethyl groups, which donate electrons to or attract unpaired electrons from free radicals, effectively trapping them. When the -COOH groups dissociate into -COO−, this electron-donating role lowers the dissociation energy of hydroxyl groups, making hydrogen atom transfer and free radical scavenging more efficient [84]. Carboxymethylation enhances the polarity and solubility of polysaccharides, significantly improving their mobility and utilization, which in turn boosts their ability to scavenge free radicals. Carboxymethylated polysaccharides can also inhibit the generation of hydroxyl radicals by chelating transition metal ions, exerting their antioxidant effects indirectly [85].

In a recent study by Liu et al., polysaccharides were isolated and purified from *Craterellus cornucopioides* (CCPs-1 and CCPs-2). To enhance their antioxidant activity, two carboxymethylated polysaccharides, cmCCPs-1 and cmCCPs-2, were created with degrees of substitution of 0.34 and 0.52, respectively. cmCCPs-2 exhibited the highest activity in scavenging DPPH radicals (EC50 = 136.0 μg/mL), while CCPs-1 showed the lowest scavenging activity (EC50 = 233.2 μg/mL). These results suggest that chemically modified polysaccharides have advantageous free radical scavenging activity. This may be because carboxymethylated polymers have a larger surface area and are more soluble in water, which makes it easier for them to interact with free radicals and boost antioxidant activity [86].

After sulfate modification, the presence of sulfated groups significantly impacts the antioxidant activity of natural polysaccharides. The higher activation capacity of the sulfated groups enhances their ability to donate hydrogen atoms or anionic charges, thereby boosting the antioxidant activity [83]. Additionally, sulfated polysaccharides may possess more surface voids, increasing their contact area with free radicals in solution. This improved interaction leads to more efficient scavenging of free radicals, resulting in stronger antioxidant activity compared with unmodified polysaccharides [87]. In a study, polysaccharides extracted from *Cyclocarya paliurus* were chemically modified to produce sulfated derivatives. The results confirm the success of the sulfation modifications, demonstrating significant changes in the physicochemical and antioxidant properties of the polysaccharides post-modification. S-CP1-8, the sulfated polysaccharide with the highest protein content and the lowest molecular weight and degree of substitution, demonstrated the best antioxidant properties in every test. These findings suggest that the enhanced in vitro antioxidant activities of the sulfated polysaccharides from *Cyclocarya paliurus* are likely influenced by a combination of factors including degree of substitution, molecular weight, sulfate content, and protein content [88].

In another study, researchers enhanced the *Tremella fuciformis* polysaccharide (TFP) by grafting it with catechin and assessed its antioxidant activity using the DPPH radical scavenging assay. They found that the catechin-grafted TFP demonstrated significantly higher antioxidant activity than the unmodified TFP. This suggests that grafting TFP with catechin can boost its antioxidant properties, making it a promising candidate for use as a novel antioxidant in the food and pharmaceutical industries [89].

### 5.2. Immunomodulatory Properties

These biopolymers have garnered significant attention due to their diverse biological activities, among which their immunomodulatory properties stand out. Immunomodulation refers to the modification of the immune response or the functioning of the immune system, which can either enhance (immunostimulation) or suppress (immunosuppression) the immune responses. Natural polysaccharides possess significant immunomodulatory properties, making them valuable in the development of therapeutic agents for various immune-related conditions [90]. Their ability to modulate the immune system through different mechanisms highlights their potential to enhance health and combat diseases [91,92].

Numerous research studies published recently have shown that some polysaccharides that have undergone chemical modification have better biological activity than the corresponding native polysaccharides [93,94]. The report indicates that *Abrus cantoniensis*, recognized as a traditional Chinese health product, demonstrates significant immunomodulatory properties attributed to its polysaccharide components. Additionally, the carboxymethylation of these polysaccharides can enhance their biological activity. This study examines how carboxymethylated *Abrus cantoniensis* polysaccharide (CM-ACP) influences immunosuppression and intestinal damage resulting from cyclophosphamide treatment in mice. The results show that when CM-ACP was compared with ACP, it had a stronger immunomodulatory effect. Furthermore, in immunosuppressed mice, CM-ACP efficiently increased the number of bacteria that produce short-chain fatty acids (SCFAs) and controlled the expression of genes that mediate signal transducer and activator of transcription 6 (STAT6) and STAT3-mediated pathway signals [95]. Research shows that sulfated polysaccharides demonstrate the capacity to exert immunomodulatory effects. Assef et al. assessed the immunostimulant efficacy of Sulfated *Dictyota caribaea* (DcSp) using a mouse Mfs cell line (RAW 264.7). *Dictyota caribaea* is a polysaccharide obtained from brown algae. Results show that, in RAW 264.7 cells, DcSp elicited immunological responses and led to the generation of M1 macrophage markers, including nitric oxide, inducible nitric oxide synthase, Interleukin-1 beta, and tumor necrosis factor-alpha [96].

### 5.3. Anticancer Properties

Cancer remains one of the leading causes of death worldwide, with projections indicating that over 35 million new cases will be diagnosed by 2050, a 77% increase from the estimated 20 million cases in 2022 [97]. This alarming rise in cancer incidence is driven by factors such as population aging, growth, and changes in exposure to risk factors linked to socioeconomic development [98]. Currently, surgery, chemotherapy, radiation therapy, immunotherapy, and targeted therapy are used to treat cancer at various stages. However, these treatments often come with significant drawbacks and side effects, which can adversely affect the lives of cancer patients [99]. In light of this, there is an urgent need for effective treatment strategies. Therefore, one of the major challenges for researchers is developing new non-toxic drug molecules for cancer therapy. It has been demonstrated that polysaccharides are a powerful anti-tumor agent that targets several cancer pathways and has pleiotropic and synergistic effects when used in conjunction with chemotherapy medications [100]. The anti-cancer potential of polysaccharides was initially identified by Nauts et al. in 1946, who discovered that certain polysaccharides had the ability to induce complete remission in cancer patients [101]. New research studies suggest that some naturally occurring polysaccharides have the ability to strengthen the immune system and exhibit anti-tumor or anti-cancer properties [102,103]. Studies demonstrate the anti-tumor properties of certain polysaccharides and their derivatives, which occur through the induction of cell death and cell cycle arrest. Kokoulin et al. reported the anticancer activity of sulfated O-polysaccharide from the marine bacterium *Cobetia litoralis* KMM 3880T. This study aimed to compare the anticancer effects of sulfated lipopolysaccharides (LPS) and sulfated O-antigenic polysaccharides of LPS or modified polysaccharide (DPS) derived from *C. litoralis* KMM 3880T on two human cancer cell lines: colorectal carcinoma HTC-116 and malignant melanoma SK-MEL-28. HTC-116 and SK-MEL-28 cell lines were treated with LPS and DPS at concentrations ranging from 6.3 to 100 µg/mL and incubated for two weeks. The effects of LPS and DPS on colony formation were assessed to determine their relative anticancer efficacy. LPS was found to be more effective than DPS in suppressing colony formation. This suggests that LPS may have a stronger effect on colorectal cancer cells. DPS demonstrated a superior inhibitory effect compared with LPS. The similarity in the inhibition concentration of DPS across both cell lines suggests that DPS may have a broader range of action but with varying effectiveness depending on the cancer cell type. The comparative analysis reveals that LPS and DPS have distinct anticancer effects on HTC-116 and SK-MEL-28 cell lines. LPS was more effective against colorectal cancer cells, whereas DPS was more effective against melanoma cells [104]. In a recent study, researchers investigated the impact of ionic group introduction and protein content on the antitumor properties of polysaccharide-protein complexes. Two water-soluble polysaccharide-protein complexes, TM1 and TM2, extracted from *Pleurotus tuber-regium*, were modified to produce sulfated and carboxymethylated derivatives. The researchers modified TM1 and TM2 to obtain sulfated (S-TM1 and S-TM2) and carboxymethylated derivatives. They then assessed the in vitro antitumor activity of these derivatives by measuring their inhibition of Sarcoma 180 tumor cell proliferation at various concentrations. The antitumor activities of the sulfated and carboxymethylated derivatives were compared with those of a known chemotherapeutic agent, 5-fluorouracil. The sulfated derivatives showed notable antitumor effects. At high doses, S-TM1 achieved a 51.2% inhibition rate, while S-TM2 reached a 43.2% inhibition rate. These effects were slightly superior to those of 5-fluorouracil. Although the carboxymethylated derivatives also exhibited antitumor activity, their efficacy was generally lower compared with the sulfated derivatives. This suggests that the introduction of sulfate groups might be more effective in enhancing the antitumor properties of polysaccharide-protein complexes [105]. In the study, the authors addressed the challenge of using traditional fungal β-glucans, which typically have high molecular weights and poor water solubility, by developing water-soluble β-glucans through controlled degradation and sulfated modification from *Saccharomyces cerevisiae*. They successfully produced a sulfated derivative (SCGS) with a molecular weight of 146 kDa and a sulfate degree of 2.04. According to the study, SCGS has the potential to be a novel anticancer drug since it binds well to fibroblast growth factor (FGF) and vascular endothelial growth factor (VEGF), but natural β-glucan does not. Additionally, by examining SCGS’s influence on the migration of highly metastatic melanoma B16F10 cells, the investigators assessed the functional impact of the treatment and discovered a considerable suppression. With an in vivo inhibition rate of 86.7%, SCGS significantly decreased and eradicated metastatic nodules in a lung metastasis mouse model. Interleukin-1 beta (IL-1β), tumor necrosis factor-alpha (TNF-α), and interferon-alpha (IFN-α) levels all showed a significant improvement. The scientists ascribed the anti-tumor and anti-metastasis mechanisms of SCGS to its capacity to control the release of angiogenic factors, preserve the extracellular matrix, and regulate degrading enzymes. Their findings laid the groundwork for exploring SCGS in the development of new anti-tumor and anti-metastasis drugs and opened new avenues in cancer research [106].

### 5.4. Anticoagulant Properties

**Figure 8:**
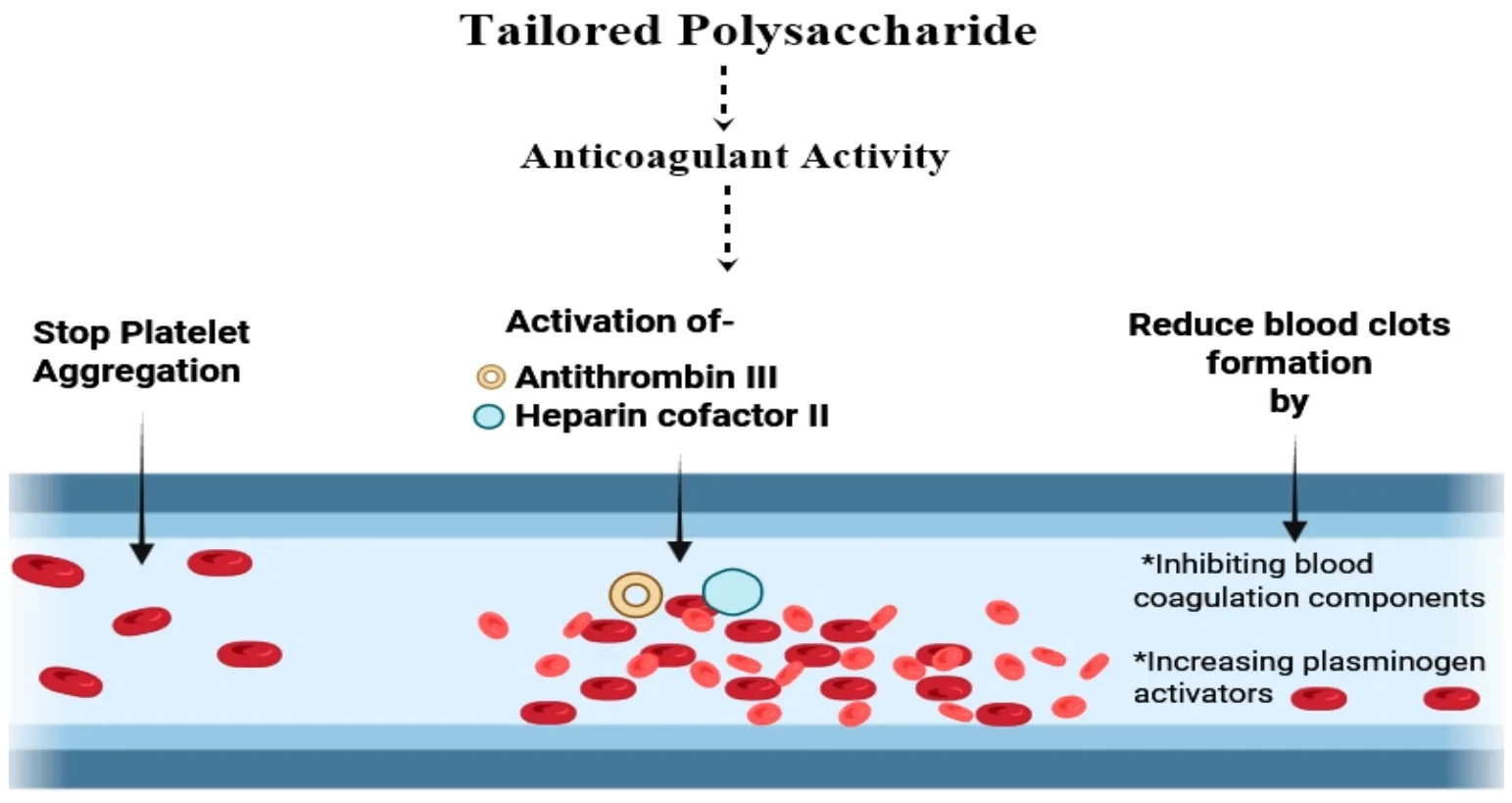
Mechanisms of anticoagulant activity of tailored polysaccharides—illustrating their ability to inhibit platelet aggregation, activate natural anticoagulants (antithrombin III and heparin cofactor II), and reduce clot formation by blocking coagulation factors and enhancing plasminogen activation. * Main mechanisms that lead to a reduction in blood clot formation.

Anticoagulants are medications that help prevent blood clots from forming. They are commonly prescribed to people at risk of developing conditions such as deep vein thrombosis, pulmonary embolism, and stroke, especially those with atrial fibrillation or certain types of heart valve disease. Heart and blood vessel disorders, which can lead to thrombosis, are currently one of the main causes of death [107]. This is why researchers are currently working to create targeted, effective antithrombotic drugs. Anticoagulant medication is necessary for the majority of thromboembolic disorders; heparin has been in use for more than 50 years. Heparin is a naturally occurring heteropolysaccharide that has anticoagulant properties and is found in the tissues of mammals. Due to heparin’s negative effects and side effects, including bleeding, chemical heterogeneity, fluctuations in physiological activity, and the possibility of infection with highly contagious agents like bovine spongiform encephalopathy, alternatives to the drug have drawn a lot of attention in research. The anticoagulant effect of modified polysaccharides is a promising area of research. Tailored polysaccharides work as anticoagulants through a variety of mechanisms that work together to stop blood clots from forming and spreading (Figure 8). First of all, they stop platelets from clumping together and forming a thrombus (clot) by inhibiting platelet aggregation. Second, they trigger the activation of natural anticoagulant proteins, including heparin cofactor II and antithrombin III, which in turn deactivate important coagulation cascade enzymes, especially thrombin (factor IIa) and factor Xa. Furthermore, polysaccharides reduce the formation of clots by directly inhibiting blood coagulation components, such as the prothrombinase complex. Lastly, by increasing the activity of plasminogen activators, which break down fibrin clots, they support the fibrinolytic system. Polysaccharides exhibit strong anticoagulant potential through this multi-targeted strategy, making them attractive treatment options for thrombotic diseases.

Modified polysaccharides, particularly sulfated polysaccharides (including heparan sulfate, dermatan sulfate (DS), chondroitin sulfate, hyaluronan, fucan sulfate, chitosan sulfate, dextran sulfate, and sulfated mannan) [108], have shown potential as alternatives to traditional anticoagulants like heparin [109]. These compounds can inhibit blood clot formation by interfering with the coagulation cascade, similar to heparin but with potentially fewer side effects [110]. Some sulfated polysaccharides enhance the activity of antithrombin [107]. The introduction of sulfate groups can enhance the anticoagulant activity of polysaccharides by increasing their negative charge density, thereby promoting interactions with coagulation-related proteins. In particular, sulfated polysaccharides derived from *Zingiber officinale* exhibited stronger anticoagulant activity when characterized by a higher degree of sulfation and an appropriate molecular weight. These findings suggest that both the extent of sulfation and molecular weight are important structural determinants of the anticoagulant activity of modified polysaccharides [111].

In a recent investigation by Liu et al., the impact of carboxymethylation and phosphorylation modifications on *Sepia esculenta* ink polysaccharide (SIP) was explored. SIP, a glycosaminoglycan sourced from *Sepia esculenta* ink, has demonstrated diverse functionalities in marine materials, including antitumor, antioxidation, immunoregulation, and chemoprevention. The study revealed that carboxymethylation and phosphorylation significantly enhanced the anticoagulant properties of SIP, leading to prolonged activated partial thromboplastin time, prothrombin time, and thrombin time. Phosphorylated polyanions have been shown to exert anticoagulant effects by promoting the interaction between heparin cofactor II and thrombin, with the binding primarily influenced by negative charges and polymeric characteristics. Authors concluded that carboxymethylation exhibited greater efficacy in enhancing SIP’s anticoagulant capacity compared with phosphorylation [112].

### 5.5. Anti-Inflammatory Properties

**Figure 9:**
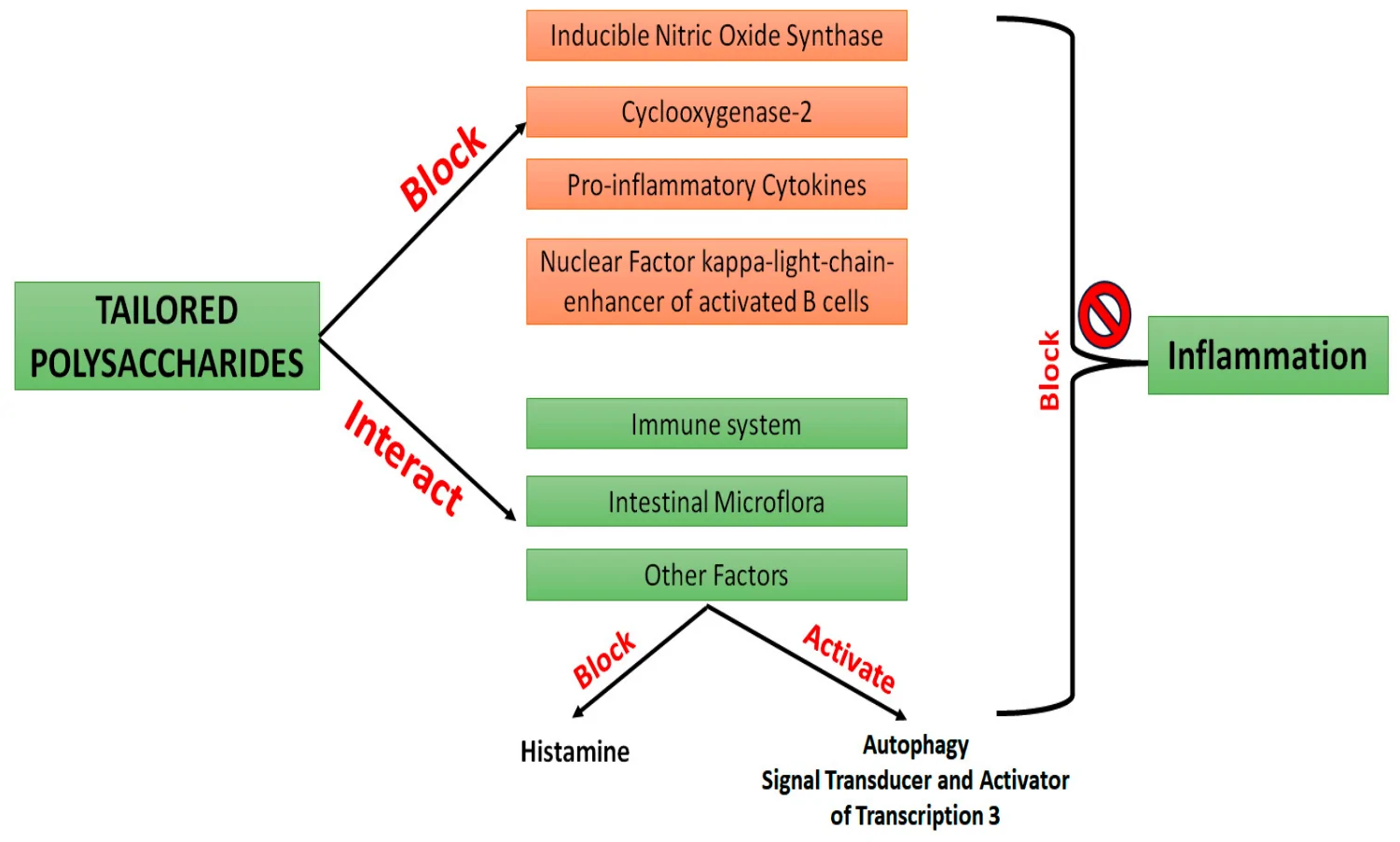
A schematic representation of the anti-inflammatory mechanism of tailored polysaccharides.

Injury, infection, and stress trigger the body’s immune system to respond to inflammation [113]. Many diseases, including arthritis, atherosclerosis, and cancer, are closely linked to chronic inflammation [114]. The conventional approach to treating inflammation relies heavily on steroid and non-steroidal anti-inflammatory drugs, both of which come with increasingly noticeable side effects that cannot be overlooked. Polysaccharides derived from natural sources have garnered significant interest in recent years due to their safety, availability, and powerful anti-inflammatory properties [115]. Various polysaccharides, including starch, glucan, gum acacia, inulin, and pectin, have been documented to exhibit anti-inflammatory properties [116]. Polysaccharides have a wide range of anti-inflammatory effects, including inhibition of pro-inflammatory mediators, immunological modulation, antioxidant activity, interaction with gut bacteria, interference with cell signaling pathways, and barrier function augmentation. These mechanisms highlight their efficacy as therapeutic agents for inflammatory disorders [117]. Figure 9 shows the pathway of the anti-inflammatory effect of polysaccharides. These compounds block inflammatory mediators such as inducible nitric oxide synthase, cyclooxygenase-2, pro-inflammatory cytokines, and nuclear factor kappa-light-chain-enhancer of activated B cells while interacting with the immune system, intestinal microflora, and other biological factors. Tailored polysaccharides also block histamine and activate autophagy and signal transducer and activator of transcription-3 pathways, collectively contributing to the inhibition of inflammation.

The anti-inflammatory activities of polysaccharides depend on their chemical structure, including compositions of monosaccharides, molecular weights, chain conformations, glycosidic connections, and sulfate concentration [117]. Adding sulfated groups to polysaccharides enhances their bioactivity, including anti-inflammatory properties [83,113]. Sulfated polysaccharides can ameliorate macrophage inflammation by lowering phagocytosis and nitric oxide generation [118]. Sulfated fungal polysaccharides from *Antrodia cinnamomea* significantly inhibited TNF-α and IL-6 production compared with native polysaccharides. Wang et al. studied the impact of sulfated modification on the anti-inflammatory property of *Cyclocarya paliurus* polysaccharides. Sulfated Cyclocarya paliurus polysaccharides CP (S-CP1-4) were produced via the chlorosulfonic acid–pyridine technique. In lipopolysaccharide-treated macrophages (RAW 264.7 cells), S-CP1-4 reduced nitric oxide generation, phagocytic activity, and the release of IL-6 and IL-1β. In lipopolysaccharide-treated mice, S-CP1-4 decreased IL-6 and TNF-α secretion, thymus and spleen indices, and boosted IL-10 production. Thus, the authors reported that S-CP1-4 showed good anti-inflammatory activities [119].

### 5.6. Antiviral Property

The lack of effective antivirals is a major issue in healthcare globally, contributing to an increase in viral infections across all infectious disorders. In December 2019, a novel coronavirus caused pneumonia in Wuhan, Hubei Province, China. The virus soon spread globally, resulting in a pandemic [120]. Many commonly used antivirals have limited antiviral effectiveness and a narrow spectrum of action, leading to virus resistance. Most antiviral drugs have severe negative effects. Consequently, it is crucial to find antiviral substances that may successfully prevent the spread of a wide range of human viruses and to produce novel medications based on these antivirals. Polysaccharides are known for their low toxicity and minimal side effects. They have been extensively studied for their effectiveness in inhibiting various viruses, including human immunodeficiency virus (HIV) [121], duck hepatitis virus [122], herpes simplex virus [123], and canine parvovirus [124]. Modification of polysaccharides can enhance the antiviral properties of native polymers by altering their molecular weight, functional groups, charge density, branching pattern, and three-dimensional conformation [37]. Sulfated polysaccharides are widely investigated for their potent and diverse antiviral properties. Promising antiviral activities have been demonstrated by modified sulfated polysaccharides, especially against HIV. By disrupting the initial phases of viral infection, the addition of sulfate groups can significantly increase their antiviral action. The antiviral effect is largely attributed to the high negative charge of sulfate groups, which can interfere with the interaction between viral particles and receptors present on the host–cell membrane, thereby limiting viral entry [27]. Moreover, these negatively charged groups may establish electrostatic interactions with positively charged regions of viral surface proteins, disrupting key stages of infection such as viral attachment, adsorption, and penetration into host cells [37]. The sulfated derivative of chitosan was also found to protect Vero cells from cytopathic infection by Coxsackie and Rift Valley fever RNA viruses [125].

## 6. Pharmaceutical Applications

Modified polysaccharides have gained considerable attention in pharmaceutical applications due to their excellent biocompatibility, biodegradability, low toxicity, and versatile physicochemical properties. Modification of polysaccharides enables their properties to be tailored according to specific drug delivery requirements, including solubility, swelling behavior, mucoadhesion, drug-loading capacity, and degradation rate. These characteristics make polysaccharide-based materials particularly attractive for the development of advanced drug delivery systems. A major application of tailored polysaccharides is controlled and targeted drug delivery, where they can improve drug stability, enhance bioavailability, and regulate drug release over a desired period. Functional groups introduced during modification can provide stimuli-responsive behavior, allowing drug release to be influenced by factors such as pH, temperature, enzymes, or redox conditions. Furthermore, polysaccharides can interact with specific biological tissues or receptors, facilitating site-specific delivery and potentially reducing systemic side effects. Tailored polysaccharides are also extensively investigated as carriers for nanostructured drug delivery systems, including nanoparticles, nanogels, microspheres, and hydrogels.

The degree of carboxymethylation is a critical parameter for regulating colloidal behavior and drug release under varying gastrointestinal pH conditions. A study addressed the poor gastrointestinal stability and premature release of rosmarinic acid by developing carboxymethylated guar gum-based nanoparticles as a pH-responsive delivery system [126]. The nanoparticles enhanced rosmarinic acid stability, minimized premature release, and improved intestinal bioaccessibility. Comparative evaluation of carboxymethylated guar gum systems with different degrees of carboxymethylation showed that highly substituted carboxymethylated guar gum nanoparticles exhibited greater colloidal stability and acid resistance, providing effective protection of rosmarinic acid under gastric conditions. In another study, IPN beads using sodium carboxymethyl cellulose and carboxymethyl konjac glucomannan were developed for the controlled delivery of ibuprofen [20]. The prepared beads exhibited pronounced pH-dependent swelling and drug-release behavior, with limited ibuprofen release under acidic conditions (pH 1.2) and enhanced, sustained release at pH 7.4. This behavior was attributed to the ionization of carboxyl groups within the polymer network, which promoted swelling under alkaline conditions and facilitated drug diffusion. The findings suggest that such pH-responsive polysaccharide-based systems may help minimize drug exposure in the stomach and improve controlled drug release in the intestinal environment, thereby offering a promising approach for oral delivery of drugs associated with gastric irritation. In a study, pH-responsive microspheres were developed for colon-specific delivery of phosphorylated wild ginseng polysaccharide as a potential therapeutic approach for ulcerative colitis [127]. The phosphorylated wild ginseng polysaccharide-based microspheres exhibited good stability under acidic gastrointestinal conditions while facilitating preferential release in the colonic environment. In a mouse model of ulcerative colitis, oral administration of the microspheres reduced intestinal mucosal damage and inflammatory responses while improving intestinal barrier integrity. These findings suggest that pH-responsive tailored polysaccharide-based microspheres may provide an effective strategy for targeted treatment of inflammatory disorders of the colon.

A recent study by Fahimi Kabir et al. developed an injectable dual-functional system based on sulfated carboxymethyl chitin and hyaluronic acid for osteoarthritis therapy [128]. Sulfated carboxymethyl chitin microparticles were prepared by emulsion-gelation and loaded with triamcinolone acetonide, while their interaction with hyaluronic acid produced an injectable, shear-thinning hydrogel-like viscosupplement. The system provided sustained triamcinolone acetonide release for 96–164 h, which was extended to approximately 180 h in the presence of hyaluronic acid. The formulation also demonstrated favorable biocompatibility and chondroitin sulfate-mimetic behavior, suggesting its potential to combine joint lubrication with prolonged anti-inflammatory drug delivery. Modified polysaccharides also show considerable potential for localized drug delivery. In a recent study, a phosphorylated pullulan-based delivery matrix was developed for the localized delivery to control oral biofilms [129]. Pullulan derived from the black yeast *Aureobasidium pullulans* was combined with the cationic surfactant cetylpyridinium chloride to develop a pullulan–cetylpyridinium chloride system for preventing oral infections. The formulation retained significant bactericidal activity at a cetylpyridinium chloride concentration of 0.01%, which is one-fifth of the conventional concentration. Furthermore, interactions between the formulation and hydroxyapatite suggested enhanced retention of cetylpyridinium chloride on tooth surfaces and its sustained local release. These findings demonstrate the potential of modified pullulan matrices as localized antimicrobial delivery systems for preventing dental caries and periodontal infections.

The pharmaceutical applications of tailored polysaccharides are summarized in Table 5.

## 7. Conclusions and Future Perspectives

Natural polysaccharides are abundant, renewable, biocompatible, and structurally versatile biomacromolecules with considerable potential for applications in food, pharmaceutical, biomedical, and cosmetic fields. Their intrinsic physicochemical and biological properties can be substantially tailored through chemical, physical, and enzymatic modifications. Polysaccharide reengineering is not merely a means of improving an existing material, but an effective strategy for engineering polysaccharides with application-specific functions.

A key design principle emerging from the current research is that modification should be guided by the desired function rather than by modification chemistry alone. The nature, degree, and spatial distribution of introduced functional groups; molecular weight; degree of branching and substitution; conformational changes; and interactions with drugs, proteins, cells, and other biomolecules should be considered collectively when designing reengineered polysaccharides. Importantly, the same chemical modification may produce markedly different outcomes depending on the polysaccharide backbone, reaction conditions, molecular-weight distribution, and substitution pattern. Consequently, controlling the extent and location of modification, while preserving favorable structural features of the parent polysaccharide, represents a central principle for achieving predictable functionality.

Despite substantial progress, several structure–function relationships remain unresolved. In many studies, correlations between degree of substitution or functional-group content and biological activity are reported without adequately distinguishing the contributions of molecular weight, chain conformation, charge density, branching, aggregation, and solubility. Furthermore, differences in polysaccharide source, extraction and purification procedures, molecular-weight distribution, analytical methods, and modification protocols can produce substantial batch-to-batch variability, making direct comparison between studies difficult. The biological activities of modified polysaccharides are also frequently evaluated using different experimental models and endpoints, limiting the establishment of broadly applicable structure-activity relationships.

Future research should therefore move toward predictive and standardized polysaccharide engineering. First, systematic structure–property–biofunction maps should be established using well-characterized polysaccharides with controlled molecular weight, substitution degree and pattern, branching, and charge density. Particular attention should be given to parameters such as molecular-weight distribution, degree and position of substitution, purity, residual reagents, structural heterogeneity, and batch-to-batch variation.

A further priority is the development of sustainable and scalable modification technologies. Enzymatic and other selective approaches, solvent-minimized processes, and green-chemistry strategies could reduce the environmental burden associated with conventional chemical modification while providing improved control over reaction selectivity. Additionally, the integration of polysaccharide-based systems with emerging technologies like nanotechnology and 3D printing could lead to the creation of highly specialized drug-delivery platforms. These platforms could offer personalized medicine solutions, where drug delivery systems are customized to the unique needs of individual patients, optimizing therapeutic outcomes.

Looking forward, the evolution of polysaccharide reengineering will be heavily driven by precision site-selective modification techniques and the design of stimuli-responsive ‘smart’ drug-delivery systems. Furthermore, integrating computational modeling, molecular dynamics, and artificial intelligence/machine learning algorithms will accelerate the transition from trial-and-error chemistry to rational, predictive materials design, laying the foundation for customizable and clinically viable biopolymer platforms.

From a translation perspective, successfully bridging the gap between laboratory synthesis and clinical or commercial implementation requires overcoming key operational hurdles. Issues surrounding industrial scalability, batch-to-batch reproducibility, long-term safety, and strict regulatory considerations must be rigorously addressed alongside material design.

Overall, the principal limitation of the current field is not a lack of available modification strategies, but the limited ability to predict how a specific structural modification will translate into a defined physicochemical or biological outcome. Future progress should therefore prioritize rational, selective, and reproducible modification over the simple expansion of chemical diversity. The next generation of polysaccharide reengineering should establish quantitative structure–property–biofunction relationships, supported by standardized characterization, mechanistic studies, computational prediction, sustainable manufacturing, and rigorous biological validation. Such an integrated approach will enable the transition from empirical modification of natural polysaccharides toward predictive materials design, ultimately facilitating the development of safer, more reproducible, application-specific polysaccharide platforms for advanced drug delivery and biomedical applications.

## Figures and Tables

**Table 1:** Search string for PubMed, Scopus, and Web of Science.

Database	Search String
PubMed	(“Polysaccharides”[Mesh] OR polysaccharide*[Title/Abstract]) AND (“polysaccharide modification”[Title/Abstract] OR “chemical modification”[Title/Abstract] OR “physicochemical modification”[Title/Abstract] OR carboxymethylat*[Title/Abstract] OR “sulfated polysaccharide*”[Title/Abstract] OR sulfation[Title/Abstract] OR phosphorylat*[Title/Abstract] OR graft*[Title/Abstract] OR “grafting polysaccharide*”[Title/Abstract] OR “interpenetrating polymer network*”[Title/Abstract] OR IPN[Title/Abstract]) AND (antioxidant*[Title/Abstract] OR anticancer[Title/Abstract] OR “anti-cancer”[Title/Abstract] OR anticoagulant*[Title/Abstract] OR immunomodulat*[Title/Abstract] OR antiviral[Title/Abstract] OR “anti-viral”[Title/Abstract] OR anti-inflammatory[Title/Abstract] OR “drug delivery”[Title/Abstract] OR pharmaceutical*[Title/Abstract] OR pharmacological*[Title/Abstract])
Scopus	TITLE-ABS-KEY(polysaccharide*) AND TITLE-ABS-KEY(“polysaccharide modification” OR “chemical modification” OR “physicochemical modification” OR carboxymethylat* OR “sulfated polysaccharide*” OR sulfation OR phosphorylat* OR graft* OR “grafting polysaccharide*” OR “interpenetrating polymer network*” OR IPN) AND TITLE-ABS-KEY(antioxidant* OR anticancer OR “anti-cancer” OR anticoagulant* OR immunomodulat* OR antiviral OR “anti-viral” OR anti-inflammatory OR “drug delivery” OR pharmaceutical* OR pharmacological*)
Web of Science	TS=(polysaccharide*) AND TS=(“polysaccharide modification” OR “chemical modification” OR “physicochemical modification” OR carboxymethylat* OR “sulfated polysaccharide*” OR sulfation OR phosphorylat* OR graft* OR “grafting polysaccharide*” OR “interpenetrating polymer network*” OR IPN) AND TS=(antioxidant* OR anticancer OR “anti-cancer” OR anticoagulant* OR immunomodulat* OR antiviral OR “anti-viral” OR anti-inflammatory OR “drug delivery” OR pharmaceutical* OR pharmacological*)

**Table 2:** Some important natural polysaccharides and their respective source and pharmaceutical applications.

Polysaccharide	Scientific/Biological Source	Family/Phylum	Category	Pharmaceutical Applications
Cellulose	*Gossypium hirsutum* (cotton); *Populus* spp. (wood pulp)	Malvaceae; Salicaceae	Plant	Tablet diluent/binder (microcrystalline cellulose); capsule excipient; controlled-release matrices; wound dressings; cellulose derivatives for film coating
Gum Arabic	*Acacia senegal* (L.) Willd.	Leguminosae (Fabaceae)	Plant	Tablet binder; emulsifier/stabilizer; suspending agent; encapsulation of drugs and flavors
Locust Bean Gum	*Ceratonia siliqua* L.	Fabaceae	Plant	Thickening/suspending agent; controlled-release formulations; matrix former; pharmaceutical gels
Guar Gum	*Cyamopsis tetragonoloba* (L.) Taub.	Leguminosae	Plant	Thickener; tablet binder; controlled-release drug-delivery matrix; mucoadhesive formulations
Karaya Gum	*Sterculia urens* Roxb.	Sterculiaceae	Plant	Bulk-forming laxative; suspending/thickening agent; tablet binder; controlled-release and mucoadhesive formulations
Konjac Glucomannan	*Amorphophallus konjac* K. Koch	Araceae	Plant	Controlled-release matrix; gelling/thickening agent; drug-delivery carrier; dietary fiber/laxative preparations
Okra Gum	*Abelmoschus esculentus* (L.) Moench	Malvaceae	Plant	Tablet binder; suspending agent; mucoadhesive drug delivery; controlled-release formulations
Tamarind Seed Polysaccharide	*Tamarindus indica* L.	Leguminosae	Plant	Ophthalmic formulations as viscosity enhancer; sustained/controlled-release systems; mucoadhesive drug delivery
Ispaghula Mucilage	*Plantago ovata* Forssk.	Plantaginaceae	Plant	Bulk-forming laxative; suspending agent; controlled-release matrix; colon-targeted drug delivery
Aloe Mucilage	*Aloe barbadensis* Mill.	Asphodelaceae	Plant	Topical preparations; wound-healing/hydrogel formulations; moisturizing and soothing pharmaceutical preparations
Pectin	*Citrus sinensis*; *Malus domestica*	Rutaceae; Rosaceae	Plant	Gelling/thickening agent; suspending agent; controlled-release matrix; colon-targeted drug delivery
Starch	*Zea mays*; *Solanum tuberosum*	Poaceae; Solanaceae	Plant	Tablet diluent, binder and disintegrant; capsule excipient; controlled-release matrix; absorbent
Chitin	Exoskeleton of *Penaeus vannamei* (shrimp); *Callinectes sapidus* (crab)	Crustacea (Malacostraca)	Animal	Wound dressings; drug-delivery systems; tissue-engineering scaffolds; hemostatic materials
Chitosan	Deacetylated chitin; crustacean/fungal sources	Crustacea; Zygomycota	Animal/ Fungal	Mucoadhesive drug delivery; nanoparticles/microparticles; wound dressings; antimicrobial formulations; controlled/targeted drug delivery
Hyaluronic Acid	Extracellular matrix of *Homo sapiens*; rooster comb (*Gallus gallus*)	Mammalia; Aves	Animal	Ophthalmic preparations; intra-articular injections; wound healing; dermal fillers; drug-delivery and tissue-engineering systems
Chondroitin Sulfate	Bovine/porcine cartilage (*Bos taurus*; *Sus scrofa*)	Mammalia	Animal	Osteoarthritis preparations; joint-support formulations; drug-delivery systems; tissue-engineering applications
Dermatan Sulfate	Porcine skin/intestinal mucosa	Mammalia	Animal	Anticoagulant research; drug-delivery systems; biomaterials and tissue-engineering applications
Heparin	Porcine intestinal mucosa (*Sus scrofa*)	Mammalia	Animal	Anticoagulant; prevention/treatment of venous thromboembolism; prevention of clotting during extracorporeal circulation and procedures
Dextran	*Leuconostoc mesenteroides*; *Streptococcus mutans*	Lactobacillaceae; Streptococcaceae	Microbial	Plasma volume expander; anticoagulant adjunct; pharmaceutical stabilizer; drug-delivery carrier
Xanthan Gum	*Xanthomonas campestris*	Xanthomonadaceae	Microbial	Suspending/thickening agent; ophthalmic viscosity enhancer; oral liquid formulations; controlled-release matrix
Gellan Gum	*Sphingomonas elodea* (formerly *Pseudomonas elodea*)	Sphingomonadaceae	Microbial	In situ gelling systems; ophthalmic drug delivery; oral controlled-release formulations; suspending agent
Bacterial Alginate	*Pseudomonas aeruginosa*; *Azotobacter vinelandii*	Pseudomonadaceae	Microbial	Encapsulation and controlled drug delivery; wound dressings; tissue-engineering biomaterials
Agar-Agar	*Gelidium amansii*; *Gracilaria* spp.	Gelidiaceae; Gracilariaceae	Algal	Suspending/gelling agent; suppository base; culture media; controlled-release and hydrogel drug-delivery systems
Alginate	*Macrocystis pyrifera*; *Laminaria hyperborea*; *Ascophyllum nodosum*	Lessoniaceae; Laminariaceae	Algal	Wound dressings; gastric-retentive and controlled-release systems; microencapsulation; dental impressions; antacid formulations
Carrageenan	*Kappaphycus alvarezii* (formerly *Eucheuma cottonii*); *Eucheuma spinosum*	Solieriaceae	Algal	Thickening/suspending agent; topical gels; controlled-release matrices; mucoadhesive and hydrogel drug-delivery systems

**Table 3:** Advantages and disadvantages of several common methods for polysaccharide modification and key outcome parameters.

Modification Method	Subtype	Advantages	Disadvantages	Production & Scalability	Safety Considerations	Key Outcome Parameter	Translational Limitations	References
Carboxymethylation	—	Simple, scalable, and cost-effective; well-characterized reaction kinetics; compatible with aqueous reaction media	Requires alkaline conditions; uneven degree of substitution (DS) distribution may occur; reduction in thermal stability	High: readily adaptable to larger-scale processing; established industrial chemistry	Residual alkali, solvents, and reaction reagents must be controlled; adequate purification required	Degree of substitution (DS); water solubility	Batch-to-batch variation in DS and starting-material composition; biological performance may depend strongly on DS	[61]
Sulfation	Chlorosulfonic acid–pyridine	High DS achievable; reproducible; suitable for diverse polysaccharides	Chlorosulfonic acid (CSA) is moisture-sensitive and potentially explosive; pyridine is toxic and malodorous; strict anhydrous conditions required	Moderate: scale-up requires strict control of moisture, temperature, and reagent handling	Corrosive reagents, toxic pyridine, hazardous reaction products; residual sulfur-containing reagents require control	DS; activated partial thromboplastin time (APTT) prolongation; DPPH EC₅₀	Reagent toxicity, purification requirements, structural heterogeneity, and regulatory concerns may limit pharmaceutical translation	[23,62]
	Sulfur trioxide–pyridine	Mild and controllable reaction conditions; applicable to pharmaceutical-grade substrates	Expensive reagent; generally limited to small-scale synthesis	Moderate: technically feasible but reagent cost can limit scale	Toxic/corrosive reagent and solvent; residual pyridine must be controlled	DS; anticoagulant activity	Cost, residual solvent concerns, and scale-up reproducibility	[30]
	Concentrated sulfuric acid	Low-cost reagent; fewer side reactions under optimized conditions	Risk of polysaccharide carbonization and glycosidic bond hydrolysis under strong acidic conditions	Moderate: inexpensive but requires careful process control	Corrosive; degradation products and residual acid must be removed	DS; molecular weight loss	Difficult control of degradation and substitution homogeneity; potential reproducibility concerns	[32]
Phosphorylation	Phosphoric acid/anhydride	Low reagent cost; short process steps	High-temperature requirement promotes chain degradation; low product yield	Moderate: potentially scalable but temperature control is important	Corrosive reagents and potential residual phosphorus-containing species require control	DS; charge density	Lower reaction efficiency and possible degradation may complicate scale-up	[35,36]
	Phosphorus oxychloride	High DS achievable; rapid reaction rate	Toxic byproducts; hazardous fumes; requires inert atmosphere	Low–moderate: specialized handling and controlled atmosphere may be required	Significant chemical-safety concerns; residual reagent/byproducts must be rigorously removed	DS; anticoagulant and antiviral activity	Safety, purification, regulatory acceptance, and scale-up handling are important barriers	[37,38]
	Phosphate (STMP/STPP)	Mild reaction conditions; minimal chain degradation	Low reactivity of phosphate reagents; relatively low DS achieved	Moderate–high: favorable process conditions but reaction efficiency may be limited	Lower hazard profile than highly reactive phosphorylating reagents; residual phosphate must be controlled	DS; swelling behavior	Limited DS and dependence on substrate/reactivity may restrict functional optimization	[40,41]
Grafting	Chemical (redox)	Versatile initiator systems; applicable to a wide range of monomers	Initiator residues may affect biocompatibility; homopolymer formation may occur as a side reaction	Moderate: scalable in principle, but heat/mixing and initiator control can affect reproducibility	Residual initiators, monomers, catalysts, or crosslinkers may affect biocompatibility	Grafting efficiency; percentage conversion	Complex purification, residual monomers/initiators, structural heterogeneity, and reproducibility	[48]
	Radiation-induced	No chemical initiator residues; possibility of obtaining sterile products	Expensive equipment; radiation safety requirements; risk of backbone scission at high radiation doses	Moderate: technically scalable but capital-intensive	Radiation exposure must be controlled; degradation products require evaluation	Graft yield; molecular weight distribution	High equipment cost, dose optimization, regulatory validation, and possible polymer degradation	[50]
	Microwave-induced	Rapid process; energy-efficient; uniform energy penetration	Specialized equipment required; risk of non-uniform heating in highly viscous systems	Moderate: promising for process intensification but requires scale-up validation	Generally lower chemical exposure than some conventional processes; thermal hot spots require control	Reaction time; drug loading capacity	Scale-up and uniform energy distribution remain important challenges	[53]
	Enzymatic	Highly selective; mild reaction conditions; environmentally friendly process	High enzyme cost; slow reaction kinetics; substrate specificity limits applicability	Low–moderate: scale-up may be limited by enzyme cost and process time	Generally favorable safety profile; enzyme and reaction-component residues require control	Grafting selectivity; product purity	Cost, enzyme stability, substrate specificity, and scale-up economics	[58,59]
Interpenetrating Polymer Networks (IPN)	Semi-IPN/Full IPN	Synergistic mechanical properties; tunable swelling and drug-release profiles	Complex multi-step synthesis; possibility of incomplete crosslinking; batch-to-batch variability	Moderate: scalable in principle but requires precise control of polymer ratios and crosslinking	Crosslinkers, initiators, residual monomers, and degradation products require toxicological assessment	Swelling ratio; compressive modulus	Complex manufacturing, sterilization and stability challenges, batch reproducibility, regulatory characterization	[60]

**Table 4:** Summary of research on the effect of polysaccharide modification on their physicochemical and biological properties.

Polysaccharide Source	Modification Method	Effect on Physicochemical Properties	Biological Property	Reference
*Ganoderma atrum*	Sulfate modification	↑ significant improvement in water solubility of the derivatives in a degree of substitution-dependent manner ↓ sharp decrease in molecular weight of the derivatives	Not studied	[64]
*Pleurotus tuber-regium*	Sulfate modification	↑ Water solubility ↑ Viscosity ↓ molecular weight	↑ antitumor activities in vivo and in vitro against human hepatic cancer cell line HepG2	[65]
*Ziziphus jujuba cv. Jinsixiaozao*	Sulfate modification	↑ molecular weight increased to 3.17× 105 Da ↑ water solubility increased significantly ↓ significant decrease in viscosity	↑ hydroxyl radical scavenging activity than the unsulfated polysaccharide ↑ antibacterial activity against *Escherichia coli* and *Bacillus subtilis*	[66]
*Aegle* *marmelos*	Sulfate modification	↑ higher swelling in acidic and alkaline pH as compared with unmodified gum higher negative zeta potential, higher ↑ viscosity, work of shear, firmness, consistency, cohesiveness and index of viscosity	↑ antimicrobial activity ↑ anticoagulant activity	[67]
*Lycium barbarum Seed*	Sulfate modification	↑ thermal stability	↑ antioxidant capacity	[68]
*Lessonia trabeculata*	Sulfate modification	↑ gel properties ↑ water-holding capacity ↑ higher mechanical strength	Not studied	[69]
*Amorphophallus* *muelleri Glucomannan*	Carboxymethylation	↑ degree of substitution ↑ solubility ↓ viscosity	Not studied	[70]
*Chrysanthemum indicum*	Carboxymethylation	↑ Solubility ↓ zeta potential and particle size	↑ antioxidant and antibacterial activities. ↑ fibroblast proliferation ↑ rapid hemostasis wound healing efficiency **×** cytotoxicity	[71]
*Aureobasidium pullulans*	Carboxymethylation	↑ water solubility ↓ molecular weight ↓ thermal stability	↑ antioxidant and hypoglycemic activity	[72]
*Gastrodia elata*	Carboxymethylation	↓ molecular weight ↑ Solubility	↑ antioxidant and immunomodulatory potentials ↓ minimal cytotoxicity against RAW264.7 macrophages. ↑ significantly enhanced the secretion of TNF-α and IL-6, indicating a pronounced immunostimulatory response	[73]
*Konjac Gum*	Carboxymethylation	↑ water solubility ↑ stability ↑ gel properties; gel structure remains intact under high-temperature and high-shear conditions	Not studied	[74]
*Opuntia dillenii*	Carboxymethylation & Phosphorylation	↓ reduced total sugar content ↑ increased uronic acid content ↑ improved solubility	↑ Phosphate derivative showed higher antioxidant activity than carboxymethyl derivative across all tested dose levels	[75]
*Euphorbia trigona Mill.*	Sulphation & Phosphorylation	Not studied	↑ Phosphate derivative showed significantly enhanced antioxidant activity ↑ hypoglycemic potential due to Inhibition effects on α-amylase and α-glucosidase (sulphate derivative) ↑ Pancreatic lipase inhibition (sulphate derivative) ↑ Both showed immunomodulatory capacity	[76]
*Vigna umbellata*	Sulphation & Phosphorylation	↑ Solubility: carboxymethyl derivative > phosphate derivative ↑ Free-swelling capacity: carboxymethyl derivative> phosphate derivative ↑ Increased thermal stability and resistance to enzymatic degradation ↑ Powder compactibility	Not studied	[77]
*Loligo duvauceli*	Phosphorylation	Not studied	↑ Antibacterial inhibition against both Gram-positive bacteria (*Staphylococcus aureus*) and Gram-negative bacteria (*Escherichia coli*) ↑ antioxidant capacity ↑Anticoagulant activity	[78]
*Cordia dichotoma*	Grafting (microwave-assisted)	↑ swelling ↓ thermal stability	Not studied	[79]
*Aegle marmelos gum*	Grafting (microwave-assisted)	↑ improved swelling ↑ biodegradability ↑ stability	↑ faster wound closure and minimal scarring ↑ enhanced tissue regeneration	[80]
*oat β-glucan*	Grafting	↓ crystallinity ↓ thermal stability	↑ antioxidant activity ↑ bacteriostatic activity	[81]

**Table 5:** Pharmaceutical applications of modified natural polysaccharides.

Polysaccharide	Drug	Main Advantages	Reference
Carboxymethyl starch/alginate hydrogel beads	Doxorubicin	- Controlling the release of doxorubicin - Effective treatment of cancer	[130]
Carboxymethyl konjac glucomannan/ gelatin-based hydrogel	Epigallocatechin gallate	- High drug encapsulation efficiency - Superior biodegradability - pH sensitivity - Controlled drug delivery	[131]
Calcium crosslinked carboxymethyl tamarind kernel polysaccharide	Diclofenac sodium	- Modified release of diclofenac sodium	[132]
Lycium barbarum polysaccharides grafted with doxorubicin	Doxorubicin	- pH sensitivity - Increased in vitro cytotoxicity, particularly for the human hepatic cancer cell line HepG2	[133]
Microwave-Controlled Polyacrylamide Graft Copolymer of Tamarind Seed Polysaccharide	Ceric ammonium nitrate	- Excellent thermal stability - Swelling capacities	[134]
Glycyrrhetinic acid-cyclodextrin grafted pullulan nanoparticles	Doxorubicin	- Liver-specific drug delivery - Better anticancer therapeutic effect on tumor-bearing mice	[135]
Green synthesis of polyacrylamide grafted Neem Gum by using the Microwave-assisted technique	Ciprofloxacin hydrochloride	- Extended drug release - Enhanced gastric retention	[136]
Carboxymethyl cellulose/poly (N-isopropyl acrylamide) interpenetrating polymer network	5-fluorouracil	- Temperature/pH-responsive - Efficient drug loading - Sustained release.	[137]
Cassia tora gum and sodium carboxymethyl cellulose-based IPN	Diclofenac sodium	- pH-sensitivity - Controlled release of diclofenac sodium - IPN beads could be used to lower the drug release in acidic pH and alter its release in alkaline pH - Reduced gastrointestinal side effects of DFS	[138]
Chemically modified polysaccharide cryogels	Ibuprofen	- pH-sensitive - Non-cytotoxic - Targeted drug release	[139]
Modified chitosan and karaya gum-based polyelectrolyte complex nanocomposite	5-fluorouracil and curcumin	- Combination chemotherapy (synergistic effect of the two drugs in reducing the development of cancer cells)	[140]

## Data Availability

No new data were created or analyzed in this study. Data sharing is not applicable to this article.

## References

[1] Nanda D., Behera D., Pattnaik S.S., Behera A.K. (2025). Advances in natural polymer-based hydrogels: Synthesis, applications, and future directions in biomedical and environmental fields. Discov. Polym..

[2] Cháirez-Ramírez M.H., Díaz-Rivas J.O., Contreras-Ramírez J.I., González-Laredo R.F., Gallegos-Infante J.A. (2024). Green polymer-based materials as promising therapeutical agents for non-communicable diseases. Polym. Int..

[3] Satchanska G., Davidova S., Petrov P.D. (2024). Natural and Synthetic Polymers for Biomedical and Environmental Applications. Polymers.

[4] Sharma S., Bhende M. (2024). An overview: Non-toxic and eco-friendly polysaccharides—Its classification, properties, and diverse applications. Polym. Bull..

[5] Bhardwaj H., Khute S., Sahu R.K., Jangde R.K. (2024). Recent Advances in Water-Soluble Polymer and Polymeric Nanoparticles for Pharmaceutical Application. Curr. Nanosci..

[6] Zhang Y., Du X., Wang H., He Z., Liu H. (2021). Sacubitril-valsartan cocrystal revisited: Role of polymer excipients in the formulation. Expert Opin. Drug Deliv..

[7] Chen X., Shen M., Yu Q., Chen Y., Xie J. (2024). Recent advance in chemistry modified methods of natural polysaccharides and their applications. Trends Food Sci. Technol..

[8] Page M.J., McKenzie J.E., Bossuyt P.M., Boutron I., Hoffmann T.C., Mulrow C.D., Shamseer L., Tetzlaff J.M., Akl E.A., Brennan S.E. (2021). The PRISMA 2020 statement: An updated guideline for reporting systematic reviews. BMJ.

[9] Su L., Feng Y., Wei K., Xu X., Liu R., Chen G. (2021). Carbohydrate-Based Macromolecular Biomaterials. Chem. Rev..

[10] Lu X. (2023). Changes in the structure of polysaccharides under different extraction methods. eFood.

[11] Kabir S.F., Rahman A., Yeasmin F., Sultana S., Masud R.A., Kanak N.A., Haque P. (2022). Occurrence, distribution, and structure of natural polysaccharides. Radiation-Processed Polysaccharides: Emerging Roles in Agriculture.

[12] Mizrahy S., Peer D. (2012). Polysaccharides as building blocks for nanotherapeutics. Chem. Soc. Rev..

[13] Liang J., Zhang M., Wang X., Ren Y., Yue T., Wang Z., Gao Z. (2021). Edible fungal polysaccharides, the gut microbiota, and host health. Carbohydr. Polym..

[14] Hoque M., McDonagh C., Tiwari B.K., Kerry J.P., Pathania S. (2022). Effect of high-pressure processing on the packaging properties of biopolymer-based films: A review. Polymers.

[15] Zarrintaj P., Ganjali M.R., Salmankhani A., Mashhadzadeh A.H., Munir M.T., Salehnia F., Rezapour M., Habibzadeh S., Saeb M.R. (2023). Carboxymethylated polysaccharides in drug delivery. Tailor-Made Polysaccharides in Drug Delivery.

[16] Jiang L., Wang H., Chen Z., Wu T., Liu Y., Lai M., Yang X., Li H., Ji X. (2025). Effects of carboxymethyl modification on physicochemical properties, structure of fenugreek polysaccharide and its capabilities of aroma enhancement and moisture retention in cigarettes. Int. J. Biol. Macromol..

[17] Maciel J.S., Silva D.A., Paula H.C.B., De Paula R.C.M. (2005). Chitosan/carboxymethyl cashew gum polyelectrolyte complex: Synthesis and thermal stability. Eur. Polym. J..

[18] Assaad E., Wang Y.J., Zhu X.X., Mateescu M.A. (2011). Polyelectrolyte complex of carboxymethyl starch and chitosan as drug carrier for oral administration. Carbohydr. Polym..

[19] Wang Y., Mo Q., Li Z., Lai H., Lou J., Liu S., Mao J. (2012). Effects of degree of carboxymethylation on physicochemical and biological properties of pachyman. Int. J. Biol. Macromol..

[20] Lohani A., Saxena R., Khan S., Mascarenhas-Melo F. (2024). PH-responsive IPN beads of carboxymethyl konjac glucomannan and sodium carboxymethyl cellulose as a controlled release carrier for ibuprofen. Int. J. Biol. Macromol..

[21] Xie L., Shen M., Wang Z., Xie J. (2021). Structure, function and food applications of carboxymethylated polysaccharides: A comprehensive review. Trends Food Sci. Technol..

[22] Gupta V., Puri R., Gupta S., Jain S., Rao G.K. (2010). Tamarind Kernel Gum: An Upcoming Natural Polysaccharide. Syst. Rev. Pharm..

[23] Pandit A.P., Waychal P.D., Sayare A.S., Patole V.C., Pandit A.P. (2018). Carboxymethyl Tamarind Seed Kernel Polysaccharide Formulated into Pellets to Target at Colon. Indian J. Pharm. Educ. Res..

[24] Kaur H., Ahuja M., Kumar S., Dilbaghi N. (2012). Carboxymethyl tamarind kernel polysaccharide nanoparticles for ophthalmic drug delivery. Int. J. Biol. Macromol..

[25] Kaity S., Ghosh A. (2013). Carboxymethylation of locust bean gum: Application in interpenetrating polymer network microspheres for controlled drug delivery. Ind. Eng. Chem. Res..

[26] Berezhnaya Y.D., Kazachenko A.S., Kazachenko A.S., Malyar Y.N., Borovkova V.S. (2024). Sulfation of Various Polysaccharide Structures: Different Methods and Perspectives. Chemistry.

[27] Alfinaikh R.S., Alamry K.A., Hussein M.A. (2025). Sustainable and biocompatible hybrid materials-based sulfated polysaccharides for biomedical applications: A review. RSC Adv..

[28] Wang Z., Xie J., Shen M., Nie S., Xie M. (2018). Sulfated modification of polysaccharides: Synthesis, characterization and bioactivities. Trends Food Sci. Technol..

[29] Lu X., Mo X., Guo H., Zhang Y. (2012). Sulfation modification and anticoagulant activity of the polysaccharides obtained from persimmon (*Diospyros kaki* L.) fruits. Int. J. Biol. Macromol..

[30] Jiang C., Xiong Q., Li S., Zhao X., Zeng X. (2015). Structural characterization, sulfation and antitumor activity of a polysaccharide fraction from *Cyclina sinensis*. Carbohydr. Polym..

[31] Wang L., Li X., Chen Z. (2009). Sulfated modification of the polysaccharides obtained from defatted rice bran and their antitumor activities. Int. J. Biol. Macromol..

[32] Lu X., Xu S., Liu X., Gao P., Li D. (2024). Sulfated Modification, Structural Features, and Anti-Tumor Effects of Polysaccharides from *Medicago sativa* L. Stems. https://ssrn.com/abstract=4761735.

[33] Sun Y., Sun W., Guo J., Hu X., Gong G., Huang L., Cao H., Wang Z. (2015). Sulphation pattern analysis of chemically sulphated polysaccharide LbGp1 from *Lycium barbarum* by GC–MS. Food Chem..

[34] Chen B.J., Qiao Y.J., Yu G., Wang X., Zhang Y., Fu L.L. (2024). Sulfation, characterization, antibacterial activity, and action mechanism of rice bran polysaccharides. Food Biosci..

[35] Jiang J., Meng F.-Y., He Z., Ning Y.-L., Li X.-H., Song H., Wang J., Zhou R. (2014). Sulfated modification of longan polysaccharide and its immunomodulatory and antitumor activity in vitro. Int. J. Biol. Macromol..

[36] Laffargue T., Moulis C., Remaud-Siméon M. (2023). Phosphorylated polysaccharides: Applications, natural abundance, and new-to-nature structures generated by chemical and enzymatic functionalisation. Biotechnol. Adv..

[37] Chen L., Huang G. (2018). The antiviral activity of polysaccharides and their derivatives. Int. J. Biol. Macromol..

[38] Xia S., Zhai Y., Wang X., Fan Q., Dong X., Chen M., Han T. (2021). Phosphorylation of polysaccharides: A review on the synthesis and bioactivities. Int. J. Biol. Macromol..

[39] Compart J., Singh A., Fettke J., Apriyanto A. (2023). Customizing Starch Properties: A Review of Starch Modifications and Their Applications. Polymers.

[40] Wu D.T., Meng L.-Z., Wang L.-Y., Lv G.-P., Cheong K.-L., Hu D.-J., Guan J., Zhao J., Li S.-P. (2014). Chain conformation and immunomodulatory activity of a hyperbranched polysaccharide from *Cordyceps sinensis*. Carbohydr. Polym..

[41] Wang J., Wang Y., Xu L., Wu Q., Wang Q., Kong W., Liang J., Yao J., Zhang J. (2018). Synthesis and structural features of phosphorylated *Artemisia sphaerocephala* polysaccharide. Carbohydr. Polym..

[42] Xiong X., Huang G., Huang H. (2019). The antioxidant activities of phosphorylated polysaccharide from native ginseng. Int. J. Biol. Macromol..

[43] Jiang N., Li B., Wang X., Xu X., Liu X., Li W., Chang X., Li H., Qi H. (2020). The antioxidant and antihyperlipidemic activities of phosphorylated polysaccharide from *Ulva pertusa*. Int. J. Biol. Macromol..

[44] Li H., Feng Y., Sun W., Kong Y., Jia L. (2021). Antioxidation, anti-inflammation and anti-fibrosis effect of phosphorylated polysaccharides from *Pleurotus djamor* mycelia on adenine-induced chronic renal failure mice. Int. J. Biol. Macromol..

[45] Choudhary S., Sharma K., Sharma V., Kumar V. (2020). Grafting Polymers. Reactive and Functional Polymers Volume Two: Modification Reactions, Compatibility and Blends.

[46] Patel R., Trivedi R., Raj M., Raj L. (2025). Graft copolymerization of chitosan with various monomers: A comprehensive review. Polym. Bull..

[47] Yazdi M.K., Zarrintaj P., Ganjali M.R., Salehnia F., Rezapour M., Seidi F., Saeb M.R. (2023). Grafted polysaccharides in drug delivery. Tailor-Made Polysaccharides in Drug Delivery.

[48] Purohit P., Bhatt A., Mittal R.K., Abdellattif M.H., Farghaly T.A. (2023). Polymer Grafting and its chemical reactions. Front. Bioeng. Biotechnol..

[49] Rudzinski W.E., Dave A.M., Vaishnav U.H., Kumbar S.G., Kulkarni A.R., Aminabhavi T.M. (2002). Hydrogels as controlled release devices in agriculture. Des. Monomers Polym..

[50] Kalia S., Sabaa M.W. (2013). Polysaccharide Based Graft Copolymers.

[51] Aly A.A., El-Bisi M.K. (2018). Grafting of Polysaccharides: Recent Advances. Biopolymer Grafting: Synthesis and Properties.

[52] Gu C., Le V., Lang M., Liu J. (2014). Preparation of polysaccharide derivates chitosan-graft-poly(ɛ-caprolactone) amphiphilic copolymer micelles for 5-fluorouracil drug delivery. Colloids Surf. B Biointerfaces.

[53] Hanh T.T., Huy H.T., Hien N.Q. (2015). Pre-irradiation grafting of acrylonitrile onto chitin for adsorption of arsenic in water. Radiat. Phys. Chem..

[54] Khan M.A., Shehrzade S., Sarwar M., Chowdhury U., Rahman M.M. (2001). Effect of Pretreatment with UV Radiation on Physical and Mechanical Properties of Photocured Jute Yarn with 1,6-Hexanediol Diacrylate (HDDA). J. Polym. Environ..

[55] Pino-Ramos V.H., Ramos-Ballesteros A., López-Saucedo F., López-Barriguete J.E., Varca G.H.C., Bucio E. (2016). Radiation Grafting for the Functionalization and Development of Smart Polymeric Materials. Top. Curr. Chem..

[56] Deshayes S., Liagre M., Loupy A., Luche J.L., Petit A. (1999). Microwave activation in phase transfer catalysis. Tetrahedron.

[57] Sharma S., Parne S.R., Panda S.S.S., Gandi S. (2024). Progress in microwave absorbing materials: A critical review. Adv. Colloid Interface Sci..

[58] Singh C., Khanna V., Singh S. (2023). Sustainability of microwave heating in materials processing technologies. Mater. Today Proc..

[59] Singh B., Kumar A. (2018). Radiation-induced graft copolymerization of N-vinyl imidazole onto moringa gum polysaccharide for making hydrogels for biomedical applications. Int. J. Biol. Macromol..

[60] Muhammad Iqbal F., Ahmad M., Tulain U.R. (2017). Microwave radiation induced synthesis of hydroxypropyl methylcellulose-graft-(polyvinylalcohal-co-acrylic acid) polymeric network and its in vitro evaluation. Acta Pol. Pharm. Drug Res..

[61] Gübitz G.M., Paulo A.C. (2003). New substrates for reliable enzymes: Enzymatic modification of polymers. Curr. Opin. Biotechnol..

[62] Karaki N., Aljawish A., Humeau C., Muniglia L., Jasniewski J. (2016). Enzymatic modification of polysaccharides: Mechanisms, properties, and potential applications: A review. Enzym. Microb. Technol..

[63] Lohani A., Singh G., Bhattacharya S.S., Verma A. (2014). Interpenetrating polymer networks as innovative drug delivery systems. J. Drug Deliv..

[64] Zhang H., Wang J.Q., Nie S.P., Wang Y.X., Cui S.W., Xie M.Y. (2015). Sulfated modification, characterization and property of a water-insoluble polysaccharide from *Ganoderma atrum*. Int. J. Biol. Macromol..

[65] Tao Y., Zhang L., Cheung P.C.K. (2006). Physicochemical properties and antitumor activities of water-soluble native and sulfated hyperbranched mushroom polysaccharides. Carbohydr. Res..

[66] Feng R.F., Wang N., Kou J.-J., An X.-W., Meng F.-H., Zheng X.-J., Wang W.-W., Wang L.-L., Wang Z.-H., Liu M.-J. (2021). Sulfated Modification, Characterization and Potential Bioactivities of Polysaccharide From *Ziziphus jujuba* cv. Jinsixiaozao. Nat. Prod. Commun..

[67] Jindal M., Rana V., Kumar V., Singh R.S., Kennedy J.F., Tiwary A.K. (2013). Sulfation of *Aegle marmelos* gum: Synthesis, physico-chemical and functional characterization. Carbohydr. Polym..

[68] Zhang X.X., Zhang W.-W., Ni Z.-J., Thakur K., Zhang J.-G., Khan M.R., Xu W.-D., Wei Z.-J. (2024). Effects of different chemical modifications on physicochemical and antioxidation properties of *Lycium barbarum* seed dreg polysaccharides. Food Chem. X.

[69] Shao W., Shu Y., Wang Y., Deng Y., Zhang N., Liu X., Zhang Z., Sang Y., Yang B. (2026). Effects of sulfated *Tricholoma mongolum* Imai polysaccharides on scallop mince gel properties: Mechanisms and structural analysis. Int. J. Biol. Macromol..

[70] Yunita R., Rahman R.A., Jaafar N.R., Jailani M.N., Alewi N.A.M., Illias R.M., Yusof A.H.M., Puspaningsih N.N.T., Jaafar M.F.A., Aziz A. (2026). Effect of Degree Substitution on the Viscosity and Solubility of Carboxymethylated *Amorphophallus muelleri* Glucomannan. Malays. J. Fundam. Appl. Sci..

[71] Li X., Wu Z., Shen H., Song Q., Luo G., Qiao X., Han Y., Wu Y., Wu S., Tao Z. (2025). Carboxymethylated polysaccharides from *Chrysanthemum indicum* with antimicrobial and hemostatic properties for promoting wound healing. Int. J. Biol. Macromol..

[72] Li W., Wang M., Xu T., Zhang Y., Zhong C. (2025). Increasing Aureobasidium pullulans β-Glucan water solubility by carboxymethylation modification and its characterization study. Carbohydr. Polym. Technol. Appl..

[73] Guan H., Yin W., Zhang X., Zhao F., Cai T., Ling X. (2025). Optimization of the process of acetylation and carboxymethylation for a polysaccharide from *Gastrodia elata* and antioxidant and immunomodulatory activities test. Sci. Rep..

[74] Li Y., Guo P., Qu K., Du W., Wang Y., Chen G. (2025). Study on Carboxymethylation Modification of Konjac Gum and Its Effect in Drilling Fluid and Fracturing Fluid. Gels.

[75] Hu X., Zhu J., Yang Y., Chen X., Lou T., Zhong M., Chen Y., Huang C., Guo J., Hu Y. (2026). Ultrasonic-assisted hot water extraction of polysaccharides from *Opuntia dillenii*: Process optimization, derivatization, physicochemical properties and biological activities. Microchem. J..

[76] Zhao X., Ma Y., Wen H., Xiong L., Wang C., Zhang J., Shao P., Zhao Y., Zhang J., Zhang W. (2026). Optimization of extraction, structural modification, and bioactivity of polysaccharides from *Euphorbia trigona* Mill. and their derivatives. Prep. Biochem. Biotechnol..

[77] Kittipongpatana O.S., Trisopon K., Kittipongpatana N. (2025). Functional and Pharmaceutical Properties of Physically and Chemically Modified Rice Bean (*Vigna umbellata*) Starches. Polysaccharides.

[78] Robin N., Pitchai A., Ramasamy P. (2026). Marine-derived phosphorylated chitosan: Structural insights and bioactivities of a novel carbohydrate polymer. Carbohydr. Res..

[79] Sinha J., Mijinyawa A.H., Chowdhury F.I., Yahya M.Z.A., Singh P.K., Durga G. (2025). Microwave-assisted synthesis and swelling behavior of poly lactic acid (PLA) grafted *Cordia dichotoma* gum-based hydrogels. Chem. Phys. Impact.

[80] Gaikwad D., Shete A., Deshmukh K., Chougale R., Jadhav A. (2026). Microwave-grafted *Aegle marmelos* gum—Carbopol hydrogel for efficient wound healing. Next Mater..

[81] Luo Y., Li Y.C., Wang M., Zhou L., Meng F.B., Jiang L.S. (2023). Effects of grafting methods and raw materials on the physicochemical properties and biological activities of phenolic acids grafted oat β-glucan. Food Res. Int..

[82] Xu Y., Song S., Wei Y., Wang F., Zhao M., Guo J., Zhang J. (2016). Sulfated modification of the polysaccharide from *Sphallerocarpus gracilis* and its antioxidant activities. Int. J. Biol. Macromol..

[83] Chen Y., Zhang H., Wang Y., Nie S., Li C., Xie M. (2015). Sulfated modification of the polysaccharides from *Ganoderma atrum* and their antioxidant and immunomodulating activities. Food Chem..

[84] Tao Y., Zhang R., Yang W., Liu H., Yang H., Zhao Q. (2015). Carboxymethylated hyperbranched polysaccharide: Synthesis, solution properties, and fabrication of hydrogel. Carbohydr. Polym..

[85] Chen N., Jiang T., Xu J., Xi W., Shang E., Xiao P., Duan J.-A. (2024). The relationship between polysaccharide structure and its antioxidant activity needs to be systematically elucidated. Int. J. Biol. Macromol..

[86] Liu Y., Duan X., Zhang M., Li C., Zhang Z., Hu B., Liu A., Li Q., Chen H., Tang Z. (2021). Extraction, structure characterization, carboxymethylation and antioxidant activity of acidic polysaccharides from *Craterellus cornucopioides*. Ind. Crops Prod..

[87] Li J., Chi Z., Yu L., Jiang F., Liu C. (2017). Sulfated modification, characterization, and antioxidant and moisture absorption/retention activities of a soluble neutral polysaccharide from *Enteromorpha prolifera*. Int. J. Biol. Macromol..

[88] Xie J.H., Wang Z.-J., Shen M.-Y., Nie S.-P., Gong B., Li H.-S., Zhao Q., Li W.-J., Xie M.-Y. (2016). Sulfated modification, characterization and antioxidant activities of polysaccharide from *Cyclocarya paliurus*. Food Hydrocoll..

[89] Liu J., Meng C.G., Yan Y.H., Shan Y.N., Kan J., Jin C.H. (2016). Structure, physical property and antioxidant activity of catechin grafted *Tremella fuciformis* polysaccharide. Int. J. Biol. Macromol..

[90] Zhao Y., Yan B., Wang Z., Li M., Zhao W. (2020). Natural Polysaccharides with Immunomodulatory Activities. Mini Rev. Med. Chem..

[91] Wang M., Zhu P., Zhao S., Nie C., Wang N., Du X., Zhou Y. (2017). Characterization, antioxidant activity and immunomodulatory activity of polysaccharides from the swollen culms of *Zizania latifolia*. Int. J. Biol. Macromol..

[92] Schepetkin I.A., Quinn M.T. (2006). Botanical polysaccharides: Macrophage immunomodulation and therapeutic potential. Int. Immunopharmacol..

[93] Liu J., Luo J., Ye H., Zeng X. (2012). Preparation, antioxidant and antitumor activities in vitro of different derivatives of levan from endophytic bacterium *Paenibacillus polymyxa* EJS-3. Food Chem. Toxicol..

[94] Li W.J., Chen Y., Nie S., Xie M., He M., Zhang S., Zhu K. (2011). *Ganoderma atrum* polysaccharide induces anti-tumor activity via the mitochondrial apoptotic pathway related to activation of host immune response. J. Cell. Biochem..

[95] Hu H., Sun W., Zhang L., Zhang Y., Kuang T., Qu D., Lian S., Hu S., Cheng M., Xu Y. (2024). Carboxymethylated *Abrus cantoniensis* polysaccharide prevents CTX-induced immunosuppression and intestinal damage by regulating intestinal flora and butyric acid content. Int. J. Biol. Macromol..

[96] Assef A.N.B., Teles F.B., Cinelli L.P., Wilke D.V. (2024). Sulfated polysaccharide from brown algae *Dictyota caribaea* stimulates macrophages to an antitumoral phenotype. Bioact. Carbohydr. Diet. Fibre.

[97] Bray F., Laversanne M., Sung H., Ferlay J., Siegel R.L., Soerjomataram I., Jemal A. (2024). Global cancer statistics 2022: GLOBOCAN estimates of incidence and mortality worldwide for 36 cancers in 185 countries. CA Cancer J. Clin..

[98] Global Cancer Burden Growing, Amidst Mounting Need for Services. https://www.who.int/news/item/01-02-2024-global-cancer-burden-growing--amidst-mounting-need-for-services.

[99] Mun E.J., Babiker H.M., Weinberg U., Kirson E.D., Von Hoff D.D. (2018). Tumor-Treating Fields: A Fourth Modality in Cancer Treatment. Clin. Cancer Res..

[100] Lachance J.C., Radhakrishnan S., Madiwale G., Guerrier S., Vanamala J.K. (2020). Targeting hallmarks of cancer with a food-system-based approach. Nutrition.

[101] Guo R., Chen M., Ding Y., Yang P., Wang M., Zhang H., He Y., Ma H. (2022). Polysaccharides as Potential Anti-tumor Biomacromolecules—A Review. Front. Nutr..

[102] Liu L., Nie S., Xie M. (2016). Tumor Microenvironment as a New Target for Tumor Immunotherapy of Polysaccharides. Crit. Rev. Food Sci. Nutr..

[103] Zong A., Cao H., Wang F. (2012). Anticancer polysaccharides from natural resources: A review of recent research. Carbohydr. Polym..

[104] Kokoulin M.S., Kuzmich A.S., Kalinovsky A.I., Tomshich S.V., Romanenko L.A., Mikhailov V.V., Komandrova N.A. (2016). Structure and anticancer activity of sulfated O-polysaccharide from marine bacterium *Cobetia litoralis* KMM 3880T. Carbohydr. Polym..

[105] Tao Y., Zhang Y., Zhang L. (2009). Chemical modification and antitumor activities of two polysaccharide–protein complexes from *Pleurotus tuber-regium*. Int. J. Biol. Macromol..

[106] Li J., Zheng M., Wang Z., Liu Y., Niu Q., Zhou H., Wang D., Song J., Bi H., Guo B. (2024). Anti-tumor and anti-metastasis of water-soluble sulfated β-glucan derivatives from *Saccharomyces cerevisiae*. Carbohydr. Polym..

[107] Melo F.R., Pereira M.S., Foguel D., Mourão P.A.S. (2004). Antithrombin-mediated Anticoagulant Activity of Sulfated Polysaccharides: Different Mechanisms for Heparin and Sulfated Galactans. J. Biol. Chem..

[108] Mourão P.A.S. (2015). Perspective on the Use of Sulfated Polysaccharides from Marine Organisms as a Source of New Antithrombotic Drugs. Mar. Drugs.

[109] Athukorala Y., Jung W.K., Vasanthan T., Jeon Y.J. (2006). An anticoagulative polysaccharide from an enzymatic hydrolysate of *Ecklonia cava*. Carbohydr. Polym..

[110] Desai U.R. (2004). New antithrombin-based anticoagulants. Med. Res. Rev..

[111] Wang B., Yan L., Guo S., Wen L., Yu M., Feng L., Jia X. (2022). Structural Elucidation, Modification, and Structure-Activity Relationship of Polysaccharides in Chinese Herbs: A Review. Front. Nutr..

[112] Liu H., Li F., Luo P. (2019). Effect of Carboxymethylation and Phosphorylation on the Properties of Polysaccharides from Sepia esculenta Ink: Antioxidation and Anticoagulation in Vitro. Mar. Drugs.

[113] Cheng J.J., Chao C.H., Chang P.C., Lu M.K. (2016). Studies on anti-inflammatory activity of sulfated polysaccharides from cultivated fungi Antrodia cinnamomea. Food Hydrocoll..

[114] Kong P., Cui Z.-Y., Huang X.-F., Zhang D.-D., Guo R.-J., Han M. (2022). Inflammation and atherosclerosis: Signaling pathways and therapeutic intervention. Signal Transduct. Target. Ther..

[115] Seibel J., Molzberger A.F., Hertrampf T., Laudenbach-Leschowski U., Diel P. (2009). Oral treatment with genistein reduces the expression of molecular and biochemical markers of inflammation in a rat model of chronic TNBS-induced colitis. Eur. J. Nutr..

[116] Jin M.Y., Li M.Y., Huang R.M., Wu X.Y., Sun Y.M., Xu Z.L. (2021). Structural features and anti-inflammatory properties of pectic polysaccharides: A review. Trends Food Sci. Technol..

[117] Hou C., Chen L., Yang L., Ji X. (2020). An insight into anti-inflammatory effects of natural polysaccharides. Int. J. Biol. Macromol..

[118] Zha X.Q., Xue L., Zhang H., Asghar M., Pan L., Liu J., Luo J. (2015). Molecular mechanism of a new *Laminaria japonica* polysaccharide on the suppression of macrophage foam cell formation via regulating cellular lipid metabolism and suppressing cellular inflammation. Mol. Nutr. Food Res..

[119] Wang Z., Xie J., Yang Y., Zhang F., Wang S., Wu T., Shen M., Xie M. (2017). Sulfated *Cyclocarya paliurus* polysaccharides markedly attenuates inflammation and oxidative damage in lipopolysaccharide-treated macrophage cells and mice. Sci. Rep..

[120] Zhang G., Zhang J., Wang B., Zhu X., Wang Q., Qiu S. (2020). Analysis of clinical characteristics and laboratory findings of 95 cases of 2019 novel coronavirus pneumonia in Wuhan, China: A retrospective analysis. Respir. Res..

[121] Tangsinmankong N., Kamchaisatian W., Day N.K., Sleasman J.W., Emmanuel P.J. (2004). Immunogenicity of 23-valent pneumococcal polysaccharide vaccine in children with human immunodeficiency virus undergoing highly active antiretroviral therapy. Ann. Allergy Asthma Immunol..

[122] Ming K., He M., Su L., Du H., Wang D., Wu Y., Liu J. (2020). The inhibitory effect of phosphorylated *Codonopsis pilosula* polysaccharide on autophagosomes formation contributes to the inhibition of duck hepatitis A virus replication. Poult. Sci..

[123] Liu X.X., Wan Z.J., Shi L., Lu X.X. (2011). Preparation and antiherpetic activities of chemically modified polysaccharides from *Polygonatum cyrtonema* Hua. Carbohydr. Polym..

[124] Feng H., Fan J., Yang S., Zhao X., Yi X. (2017). Antiviral activity of phosphorylated *Radix Cyathulae officinalis* polysaccharide against Canine Parvovirus in vitro. Int. J. Biol. Macromol..

[125] Dimassi S., Tabary N., Chai F., Blanchemain N., Martel B. (2018). Sulfonated and sulfated chitosan derivatives for biomedical applications: A review. Carbohydr. Polym..

[126] Yildirim A.S., Altintaş Ö.E. (2026). PH-Responsive Colloidal Guar Gum Nanoparticles for Rosmarinic Acid Delivery: Role of the Degree of Carboxymethylation. Colloids Interfaces.

[127] Huo D.Y., Li Y., Song L., Zhang W., Li X., Zhang J., Ren S., Wang Z., Li W. (2025). Colon-Targeted Ginseng Polysaccharides-Based Microspheres for Improving Ulcerative Colitis via Anti-Inflammation and Gut Microbiota Modulation. Adv. Healthc. Mater..

[128] Kabir M.F., Mirzadeh H., Goharpey F. (2026). An injectable dual-functional biomimetic system based on carboxymethyl chitin sulfate and hyaluronic acid for viscosupplementation and sustained triamcinolone acetonide release in osteoarthritis therapy. Mater. Today Chem..

[129] Namba-Koide N., Yoshida Y., Nagaoka N., Okihara T., Kawata Y., Ito M., Ito T., Takeuchi-Hatanaka K., Shinoda-Ito Y., Omori K. (2025). Phosphorylated pullulan as a local drug delivery matrix for cationic antibacterial chemicals to prevent oral biofilm. BMC Oral Health.

[130] Fang K., Zhang Y., Yin J., Yang T., Li K., Wei L., Li J., He W. (2022). Hydrogel beads based on carboxymethyl cassava starch/alginate enriched with MgFe2O4 nanoparticles for controlling drug release. Int. J. Biol. Macromol..

[131] Wang L., Zhou N., Zheng S., Pang J. (2022). Formation of composite hydrogel of carboxymethyl konjac glucomannan/gelatin for sustained release of EGCG. Food Sci. Hum. Wellness.

[132] Dagar V., Pahwa R., Ahuja M. (2023). Preparation and Characterization of Calcium Cross-linked Carboxymethyl Tamarind Kernel Polysaccharide as Release Retardant Polymer in Matrix. Biointerface Res. Appl. Chem..

[133] Wang Y., Bai F., Luo Q., Wu M., Song G., Zhang H., Cao J., Wang Y. (2019). *Lycium barbarum* polysaccharides grafted with doxorubicin: An efficient pH-responsive anticancer drug delivery system. Int. J. Biol. Macromol..

[134] Jha S., Malviya R., Fuloria S., Sundram S., Subramaniyan V., Sekar M., Sharma P.K., Chakravarthi S., Wu Y.S., Mishra N. (2022). Characterization of Microwave-Controlled Polyacrylamide Graft Copolymer of Tamarind Seed Polysaccharide. Polym..

[135] Yang W., Zhang Y., Wang J., Li H., Yang H. (2022). Glycyrrhetinic acid-cyclodextrin grafted pullulan nanoparticles loaded doxorubicin as a liver targeted delivery carrier. Int. J. Biol. Macromol..

[136] Patil T., Pawar A., Korake S., Patil R., Pawar A., Kamble R. (2022). Green synthesis of polyacrylamide grafted Neem Gum for gastro retentive floating drug delivery of Ciprofloxacin Hydrochloride: In vitro and in vivo evaluation. J. Drug Deliv. Sci. Technol..

[137] Liu Z., Zhang S., Gao C., Meng X., Wang S., Kong F. (2022). Temperature/pH-Responsive Carboxymethyl Cellulose/Poly (N-isopropyl acrylamide) Interpenetrating Polymer Network Aerogels for Drug Delivery Systems. Polymers.

[138] Lohani A., Maurya M., Kumar S., Verma N. (2023). IPN beads prepared by tailoring of cassia tora gum and sodium carboxymethyl cellulose using Al+++ for controlled drug delivery. J. Drug Deliv. Sci. Technol..

[139] Santos A.M.S., Dias É.R., Osajima J.A., Cuevas M.d.M.O., Barud H.S., Silva-Filho E.C. (2025). Chemically modified polysaccharide cryogels and their performance in ibuprofen release. J. Mol. Liq..

[140] Anjar R., Mahadev M., Charyulu R.N., Badalamoole V. (2025). Development of a novel polyelectrolyte complex nanocomposite of modified chitosan and karaya gum for co-delivery of 5-fluorouracil and curcumin for cancer therapy. J. Mater. Chem. B.

